# Hepatic transcript profiling in beef cattle: Effects of rumen-protected niacin supplementation

**DOI:** 10.1371/journal.pone.0289409

**Published:** 2023-08-03

**Authors:** Gastón F. Alfaro, Valentino Palombo, Mariasilvia D’Andrea, Wenqi Cao, Yue Zhang, Jonathan Beever, Russell B. Muntifering, Wilmer J. Pacheco, Soren P. Rodning, Xu Wang, Sonia J. Moisá

**Affiliations:** 1 Department of Animal Sciences, University of Tennessee, Knoxville, TN, United States of America; 2 Department of Animal Sciences, Auburn University, Auburn, AL, United States of America; 3 Cooperative Extension Service, University of Kentucky, Kentucky, Lexington, United States of America; 4 Department of Poultry Sciences, Auburn University, Auburn, AL, United States of America; 5 Department of Agricultural, Environmental and Food Sciences, Università degli Studi del Molise, Campobasso, Italy; 6 Department of Pathobiology, College of Veterinary Medicine, Auburn University, Auburn, AL, United States of America; 7 HudsonAlpha Institute for Biotechnology, Huntsville, AL, United States of America; University of Illinois, UNITED STATES

## Abstract

The objective of our study was to assess the effect of rumen-protected niacin supplementation on the transcriptome of liver tissue in growing Angus × Simmental steers and heifers through RNA-seq analysis. Consequently, we wanted to assess the known role of niacin in the physiological processes of vasodilation, detoxification, and immune function in beef hepatic tissue. Normal weaned calves (~8 months old) were provided either a control diet or a diet supplemented with rumen-protected niacin (6 g/hd/d) for a 30-day period, followed by a liver biopsy. We observed a significant list of changes at the transcriptome level due to rumen-protected niacin supplementation. Several metabolic pathways revealed potential positive effects to the animal’s liver metabolism due to administration of rumen-protected niacin; for example, a decrease in lipolysis, apoptosis, inflammatory responses, atherosclerosis, oxidative stress, fibrosis, and vasodilation-related pathways. Therefore, results from our study showed that the liver transcriptional machinery switched several metabolic pathways to a condition that could potentially benefit the health status of animals supplemented with rumen-protected niacin. In conclusion, based on the results of our study, we can suggest the utilization of rumen-protected niacin supplementation as a nutritional strategy could improve the health status of growing beef cattle in different beef production stages, such as backgrounding operations or new arrivals to a feedlot.

## Introduction

Niacin, also known as Vitamin B3 or nicotinic acid, is an essential water-soluble vitamin involved in numerous metabolic functions. For example, niacin is part of the cofactors NAD^+^ and NADP^+^ in the oxidized form and NADH or NADPH in the reduced form and are present in many important enzymatic pathways across organisms. Enzymes that depend on niacin are numerous and represent an important factor in metabolism in all organisms. For example, NAD^+^ participates in approximately 400 reactions, whereas NADP^+^ in 30 different reactions [1]. The major function of NAD^+^ and NADP^+^ is to regulate cellular electron transfer reactions. Thus, NADH and NADPH are strong electron donors, participating in the maintenance of the redox status of the cell [2]. Niacin is present in feedstuff, and it can be synthesized by the ruminal microflora and in the liver from tryptophan [3]. Although, in some cases could be limiting due to the extensive use in lipid, protein, and carbohydrate metabolism as a cofactor. Since B vitamins play important roles as cofactors in lipid, protein, and carbohydrate synthesis, there may exist a need for supplementation, especially when high forage levels are included in the diet, which presents lower niacin concentration compared to concentrate feeds [4]. Niacin could be minimally absorbed at the rumen level because it usually bounds to rumen microbes; however, most of the niacin is absorbed in the small intestine [1]. In order to avoid ruminal degradation, niacin can be supplemented encapsulated in a chemical coat that helps to by-pass the rumen (RPN) [5]. Rumen-protected niacin has been widely used in dairy cattle due to its numerous metabolic advantages, such as its vasodilatory, antioxidative, and antilipolytic effect [6–8]. Positive effects were observed in high-producing dairy cows supplemented with niacin in terms of feed efficiency, fat corrected milk and milk fat [9]. Even though our group investigated the effect of supplementation with RPN on beef cattle consuming endophyte-infected tall fescue on hematological parameters [10], however little information is available about its effect on beef cattle liver transcriptome. Since niacin directly affects lipid metabolism by inhibiting lipolysis, its utilization in growing beef cattle may improve liver health status. Accordingly, research studies have focused on analyzing changes in metabolic pathways in the liver of dairy cattle [11, 12]. Therefore, the objective of our study was to identify changes at the transcript level of liver tissue of growing Angus × Simmental steers and heifers by supplementation with RPN.

## Materials and methods

### Animals and experimental design

All the procedures for our study were conducted following a protocol approved by the Institutional Animal Care and Use Committee of Auburn University (IACUC Protocol # 2019–3484). During the lactation period, animals used in our study were located at the Black Belt Research Center (32°28’16.32"N 87°13’54.12"W, Marion Junction, AL), belonging to Auburn University. At weaning, steers and heifers were relocated to the Beef Evaluation Center, Auburn University, Auburn, AL, due to the accessibility to the Calan gates system (Northwood, NH, USA). A group of 6 Angus × Simmental, weaned steers (n = 4) and heifers (n = 2) with average body weight (BW; 299 ± 7 kg) and age of 7–9 months old were randomly allocated in two groups based on dietary treatment: Rumen-protected niacin (RPN; n = 3), and Control (CTRL; n = 3). There was a steer: heifer ratio of 2:1 in all treatments (e.g., n = 2 steers and 1 heifer per treatment). After a training period of approximately ten days, animals were successfully adapted to Calan gates, utilized for ensuring the administration of RPN daily dosage per head. The diet offered was *ad libitum* Bermudagrass hay combined with a nutritional supplement composed of 1.61 kg of endophyte-free tall fescue seeds, and 1.61 kg of pellets composed of 46.5% ground corn, 46.5% soybean meal, 5% wheat middlings, and 2% soybean oil; and 0.1 kg of molasses per animal per day (S1 Table). The diet was formulated to meet nutrient requirements (NRC, 2016). Rumen-protected niacin (Anevis, QualiTech, Inc., Chaska, MN, 55318) is presented as white shard or chip shaped pellets that contains 70.0 ± 2.0% of Niacin (C_6_H_5_NO_2_) and it was supplemented following the manufacturers maximum recommended dosage, which is 6 g/hd/d. In the rumen-protected form (ANEVIS), 67% of the niacin is delivered to the small intestine and the availability of the niacin fed is approximately 30% when fed at the maximum recommended dosage according to manufacture recommendation.

### Liver biopsies

Liver samples (0.5–1 g) were obtained 30 days after the beginning of the supplementation with RPN, using a sterilized bone marrow aspiration needle (Monoject™, Dublin, Ireland) [13]. An area surrounding the 11^th^ and 13^th^ ribs was scanned by ultrasound to identify the optimal area to perform the liver biopsy. Five milliliters of Lidocaine 2% (VetOne®, Boise, ID) were injected in the selected area. The incision on the skin was performed using a sterilized scalpel blade. Each liver sample was rinsed with sterile saline, placed in a sterile 2 mL cryovial, and immediately stored in liquid nitrogen for further transportation and storage at -80°C at the Beef Nutriepigenomics Laboratory of the Department of Animal Sciences, Auburn University until further analysis.

### RNA extraction and library construction

The total RNA of liver samples was extracted using the ZYMO Quick DNA/RNA Miniprep Plus Kit (Zymo Research, CA, Catalog # D7003) with the addition of a DNAse digestion incubation time of 15min. For homogenization, tissue samples with DNA/RNA Shield were mechanically homogenized by Qiagen TissueRuptor II (Qiagen, MD). RNA concentrations were measured by Qubit fluorometer 3.0 (Thermo Fisher Scientific, MA) with Qubit RNA BR Assay Kit. All RNA samples showed RIN values greater than 8.0/10.0.

RNA sequencing libraries were constructed using the NEBNext Ultra II Directional RNA Library Prep Kit for Illumina (New England Biolabs, MA) and NEBNext Poly(A) mRNA Magnetic Isolation Module (New England Biolabs, MA), with a 1500 ng total RNA input. The concentrations and the size distribution of the libraries were checked by the LabChip GX Touch HT machine using the HT DNA NGS 3K Assay (Perkin Elmer, MA). The fragment size of final libraries ranged from 343 to 409 bp. The libraries were individually barcoded and pooled. The libraries were sequenced on an Illumina NovaSeq 6000 instrument to generate 150-nucleotide paired-end reads.

### RNA-seq analysis

A total number of 628,220,974 read pairs were generated for the six transcriptomes, with sequencing yields ranging from 75,864,914 to 108,428,902 reads per sample. The read quality was checked by FastQC v11.5 [14]. Sequencing adapter sequences and low-quality bases were trimmed using Trimmomatic v0.36 [15]. On average, 98.18% of reads survived quality filtering, and these high-quality reads were mapped to the cattle reference genome (GenBank: GCA_002263795.2) by Tophat-2.1.1 [16, 17]. The average mapping percentage is 86.76% (S2 Table). RNA concentration was 834.83 ± 135.26 ng/uL.

### Differential gene expression analysis

Gene reads counts were performed in three software packages, Cufflinks-2.2.1 [16], Bedtools -2.30.0 [18], and HT-seq [19]. The counts agreed well for > 99% gene models. A manual check was performed for the remaining gene models in Integrative Genomics Viewer to determine the correct counts [20]. DESeq2 package in R were used to normalize read counts and detect differentially expressed genes (DEGs) [21, 22] at a False Discovery Rate (FDR) threshold of 0.05. The log2 fold change (LogFC) was determined for each gene. The dataset analysed during the current study is available in the NCBI Gene Expression Omnibus https://www.ncbi.nlm.nih.gov/geo/ under accession number GSE 208241.

### Quantitative reverse-transcription PCR (qRT-PCR) validation of selected DEGs in liver samples

Concentrations of the six liver RNA samples were measured by Qubit 3.0 Fluorometer (Invitrogen, CA). Reverse transcription was carried out using the RevertAid RT kit (Thermo Scientific, MA). The input of 1 μg total RNA template was mixed with 1 μL Random Hexamer primer, 4 μL 5× Reaction Buffer, 1 μL RiboLock RNase Inhibitor (20 U/μL), 2 μL 10 mM dNTP Mix, 1 μL RevertAid RT (200 U/μL) and proper volume of nuclease-free water that raised the total volume to 20 μL. The 20-μL reaction system was incubated for 5 min at 25°C in an Eppendorf Mastercycler Pro Thermal Cycler (Eppendorf, CT), followed by 60 min at 42°C, and the reaction was terminated by heating at 70°C for 5 min. The product of the first-strand cDNA synthesis was diluted at 1:1 ratio with nuclease-free water before proceeding with qPCR. Gene-specific qPCR primers were designed using Oligo 7.0 (Molecular Biology Insights Inc., CO) and synthesized at Eurofins Genomics (see S3 Table for primer sequences). Primer specificity was confirmed by the UCSC In-Silico PCR tool. Ten DEGs were selected for qRT-PCR validation based on fold change expression (S5 Fig). They were divided into four groups depending on their optimal annealing temperature. Real-time quantitative PCR was carried out using the Luna Universal qPCR Master Mix kit (New England BioLabs, MA) on a BioRad CFX Opus 96 thermocycler (Bio-Rad Laboratories, CA) with the following conditions: an initial denaturing step at 95°C for 60 s, followed by 40 cycles of denaturation at 95°C for 15 s and extension at group-specific extension temperature for 30 s. The reaction system was 20 μL in volume, composed of 10 μL Luna Universal qPCR Mix, 0.5 μL gene-specific forward primer, 0.5 μL gene-specific reverse primer, 1 μL pre-diluted cDNA template, and 8 μL nuclease-free water. After PCR amplification, a melting curve was generated by heating from 65 to 95°C with 0.5°C increments, 3 s dwell time. Each sample had two technical replicates per gene. Relative quantification of gene expression was determined by the Cq values. The results were visualized using R (S5 Fig).

### Functional annotation of genes

Functional annotation of genes was carried out to gain insight into the underlying biology of the effect of RPN supplementation in the liver. Database for Annotation, Visualization, and Integrated Discovery (DAVID, version 6.8) [23] was used for functional annotation. DAVID assigned genes to pathways as per the Kyoto Encyclopedia of Genes and Genomes (KEGG), and determined enrichment of pathways using Fisher’s exact test [24]. In order to account for multiple testing, a Benjamini-Hochberg correction was applied [25]. A list of DEG was generated using FDR < 0.05 as a cutoff value. Pathways were deemed to be significant if they obtained a corrected *P*-value of < 0.05. Pathways specifically addressing human diseases and disorders were not included in further analysis of DAVID identified pathways, as these were not relevant to our study (Table 1).

**Table 1:** Summary of flux and impact results identified by the dynamic impact approach (DIA) based on Kyoto Encyclopedia of Genes and Genomes (KEGG) pathways databases analysis of the bovine liver transcriptome of growing beef cattle supplemented with or without RPN.

KEGG category	KEGG subcategory	Impact	Flux
Global and overview maps	Carbon metabolism	616.741994	-260.7824775
	Metabolic pathways	603.078811	-226.2043279
	Biosynthesis of amino acids	532.488557	-125.1637293
	Biosynthesis of cofactors	494.30856	-195.915676
	Fatty acid metabolism	476.889691	-307.3373675
**Metabolism**		676.408654	-311.7001188
	Metabolism of Other Amino Acids	1192.1306	-503.980759
	**Glycan Biosynthesis and Metabolism**	795.436229	-612.5510388
	**Lipid Metabolism**	704.232381	-549.4342
	Nucleotide Metabolism	653.534701	-334.3243263
	Amino Acid Metabolism	608.296694	-339.4627242
	Carbohydrate Metabolism	570.910956	-190.4287104
	Xenobiotics Biodegradation and Metabolism	509.163269	319.6926251
	Energy Metabolism	465.005066	334.6533257
Genetic Information Processing		680.499913	-206.8360643
	Replication and Repair	812.248268	-343.7425155
	Folding, Sorting and Degradation	657.625796	-201.0044656
	Transcription	615.987249	-159.510865
	Translation	559.854528	-44.25978518
**Environmental Information Processing**		960.903322	-784.6343456
	**Signaling Molecules and Interaction**	980.766095	-777.7726574
	**Signal Transduction**	965.723931	-785.6111315
	**Membrane Transport**	780.799763	-780.7997628
**Cellular Processes**		829.272424	-610.1620241
	**Cell Motility**	1044.29932	-823.5503655
	**Cellular community—eukaryotes**	1004.97235	-863.5606025
	**Transport and Catabolism**	765.983572	-486.3004307
	**Cell Growth and Death**	752.401923	-541.0458678
**Organismal Systems**		892.634439	-690.5794713
	**Aging**	1093.99505	-1057.552977
	**Immune System**	1004.17577	-902.8083205
	**Endocrine System**	939.544808	-763.9252758
	**Digestive System**	939.106527	-700.4125486
	**Development**	869.624754	-688.5287606
	**Circulatory System**	787.853847	-522.0104134
	Nervous System	744.077123	-419.7460141
	Excretory System	663.718215	-380.9141043
	Environmental Adaptation	574.101607	-111.8413431
	Sensory System	538.302721	-197.7461005

‘Impact’ represents the change in the expression of the genes belonging to a specific pathway due to the supplementation of RPN; and ‘flux’ as the report of the average direction in the expression as downregulation, upregulation, or neutral or no change. Flux represents the direction of each category and the corresponding subcategory: green color represents inhibition, whereas red color shows activation. Blue lines show the impact of each category and the corresponding subcategory (*P* value < 0.05; FDR < 0.05). Subcategories highlighted in bold met the defined cutoff criteria.

### Dynamic impact approach

We utilized the Dynamic Impact Approach (DIA) analysis for estimating the impact and flux of all the manually curated pathways associated with the KEGG database [26]. We defined the term ‘impact’ as the change in the expression of the genes belonging to a specific pathway due to the supplementation of RPN; and ‘flux’ as the report of the average direction in the expression as downregulation, upregulation, or neutral or no change. The entire dataset, including Entrez gene IDs, FDR, Fold Change (FC), and *p*-values of each treatment group (RPN and CTRL) were uploaded into DIA, and the overall cutoff was applied on FDR and *p*-value < 0.05 as the threshold (Table 1). The cutoff criteria for selecting relevant KEGG results for discussion was to consider those KEGG subcategories and KEGG pathways that met two cutoffs: a) having a value higher than 0.6 of the difference between the absolute value of flux and the impact value and, b) having an impact value greater than 50% of the maximum total impact. Almost all representative KEGG categories (‘Metabolism’, ‘Environmental information processing’, ‘Cellular processes’, and ‘Organismal system’) were impacted by RPN supplementation showing, in general, an inhibition (or down-regulation). The KEGG categories ‘Global and overview maps’ and ‘Genetic Information Processing’ did not have any significantly impacted KEGG subcategory according to our established cutoff criteria; therefore, they were not considered in the discussion.

### PANEV visualization analyses

PANEV (Pathway Network Visualizer) v.1.0 is an R (RStudio, Boston, MA) package which utilizes KEGG database to retrieve information about each KEGG pathway. This method helps to visualize the interconnection among key genes and KEGG pathways that were significantly impacted by the treatment applied (S1 to S4 Figs). PANEV analysis was performed as described in a recent publication [27].

## Results

All animals consumed the supplement composed by endophyte-free tall fescue and pellets along the study. Quantitative Reverse-Transcription PCR performed to validate RNA-seq data presented consistency in the level of expression of the analyzed genes (S5 Fig). A list of 1,192 DEGs (1,131 with entrez gene ID available) between animals that received RPN for 30 days and animal’s control were obtained (FDR ≤ 0.05). Overall, our DEG list generated a downregulated expression pattern by DIA [26]. Indeed, except for the ‘Energy Metabolism’ and ‘Xenobiotics Biodegradation and Metabolism’ KEGG subcategories, we observed the downregulation of all main KEGG subcategories (Table 1). In brief, the KEGG category “Metabolism” presented an inhibition of the KEGG subcategories ‘Lipid Metabolism’ and ‘Glycan Biosynthesis and Metabolism’ (Table 1). Within the ‘Glycan Biosynthesis and Metabolism, the ‘Other type of O-glycan biosynthesis’ KEGG pathway showed an inhibition and within the ‘Lipid Metabolism’ KEGG subcategory, the ‘Arachidonic acid metabolism’ KEGG pathway was inhibited as well (Table 2).

**Table 2:** Results of flux and impact uncovered by the Dynamic Impact Approach (DIA) based on Kyoto Encyclopedia of Genes and Genomes (KEGG) ‘Metabolism’ category database analysis of the bovine liver transcriptome of growing beef cattle with or without RPN supplementation.

KEGG category	KEGG subcategory	KEGG pathway	Impact	Flux
Metabolism	Carbohydrate Metabolism	Inositol phosphate metabolism		
Metabolism	Carbohydrate Metabolism	Glyoxylate and dicarboxylate metabolism		
Metabolism	Energy Metabolism	Oxidative phosphorylation		
**Metabolism**	**Lipid Metabolism**	**Arachidonic acid metabolism**		
Metabolism	Lipid Metabolism	Glycerophospholipid metabolism		
Metabolism	Lipid Metabolism	Glycerolipid metabolism		
Metabolism	Lipid Metabolism	Steroid hormone biosynthesis		
Metabolism	Nucleotide Metabolism	Purine metabolism		
Metabolism	Amino Acid Metabolism	Glycine, serine and threonine metabolism		
Metabolism	Amino Acid Metabolism	Alanine, aspartate and glutamate metabolism		
Metabolism	Amino Acid Metabolism	Arginine and proline metabolism		
Metabolism	Metabolism of Other Amino Acids	Glutathione metabolism		
**Metabolism**	**Glycan Biosynthesis and Metabolism**	**Other types of O-glycan biosynthesis**		
Metabolism	Glycan Biosynthesis and Metabolism	Glycosaminoglycan biosynthesis—chondroitin sulfate / dermatan sulfate		
Metabolism	Glycan Biosynthesis and Metabolism	N-Glycan biosynthesis		
Metabolism	Xenobiotics Biodegradation and Metabolism	Metabolism of xenobiotics by cytochrome P450		
Metabolism	Xenobiotics Biodegradation and Metabolism	Drug metabolism—cytochrome P450		

‘Impact’ represents the change in the expression of the genes belonging to a specific pathway due to the supplementation of RPN; and ‘flux’ as the report of the average direction in the expression as downregulation, upregulation, or neutral or no change. Flux represents the direction of each subcategory belonging to ‘Metabolism’ KEGG category: green color represents inhibition, yellow neutrality, whereas red color shows activation. Color intensity depicts flux level. Blue lines show the impact of each category and the corresponding subcategory (*P* value < 0.05; FDR < 0.05). Subcategories and pathways highlighted in bold met the defined cutoff criteria.

The three KEGG subcategories affected by RPN supplementation in the “Environmental information processing” KEGG category were: ‘Membrane Transport’, ‘Signal transduction’ and ‘Signaling molecules and interaction’. ‘Membrane Transport’ KEGG subcategory was affected as a whole, but none of its KEGG pathways satisfy our cutoff criteria; therefore, it was not analyzed. In contrast, ‘Signal transduction” KEGG subcategory presented the downregulation of the following pathways: ‘Jak-STAT signaling pathway’, ‘Hippo signaling pathway’, ‘VEGF signaling pathway’, ‘HIF-1 signaling pathway’, ‘TNF signaling pathway’, ‘Hedgehog signaling pathway’, ‘Apelin signaling pathway’, ‘Notch signaling pathway’, ‘FoxO signaling pathway’, ‘ErbB signaling pathway’, ‘PI3K-Akt signaling pathway’, ‘Rap1 signaling pathway’, ‘cAMP signaling pathway’, ‘Phospholipase D signaling pathway’, ‘cGMP-PKG signaling pathway’, ‘Sphingolipid signaling pathway’, ‘MAPK signaling pathway’, ‘mTOR signaling pathway’ and, ‘Wnt signaling pathway’. Furthermore, ‘ECM-receptor interaction’ pathway, which belongs to “Signaling molecules and interaction” KEGG subcategory, was also downregulated (Table 3).

**Table 3:** Results of flux and impact uncovered by the Dynamic Impact Approach (DIA) based on Kyoto Encyclopedia of Genes and Genomes (KEGG) ‘Environmental information processing’ pathway database analysis of the bovine liver transcriptome of growing beef cattle supplemented with RPN.

KEGG category	KEGG subcategory	KEGG pathway	Impact	Flux
Environmental Information Processing	Membrane Transport	ABC transporters		
**Environmental Information Processing**	**Signal Transduction**	**Jak-STAT signaling pathway**		
**Environmental Information Processing**	**Signal Transduction**	**Hippo signaling pathway**		
**Environmental Information Processing**	**Signal Transduction**	**VEGF signaling pathway**		
**Environmental Information Processing**	**Signal Transduction**	**HIF-1 signaling pathway**		
**Environmental Information Processing**	**Signal Transduction**	**TNF signaling pathway**		
**Environmental Information Processing**	**Signal Transduction**	**Hedgehog signaling pathway**		
**Environmental Information Processing**	**Signal Transduction**	**Apelin signaling pathway**		
**Environmental Information Processing**	**Signal Transduction**	**Notch signaling pathway**		
**Environmental Information Processing**	**Signal Transduction**	**FoxO signaling pathway**		
**Environmental Information Processing**	**Signal Transduction**	**ErbB signaling pathway**		
**Environmental Information Processing**	**Signal Transduction**	**PI3K-Akt signaling pathway**		
**Environmental Information Processing**	**Signal Transduction**	**Rap1 signaling pathway**		
**Environmental Information Processing**	**Signal Transduction**	**cAMP signaling pathway**		
**Environmental Information Processing**	**Signal Transduction**	**Phospholipase D signaling pathway**		
**Environmental Information Processing**	**Signal Transduction**	**cGMP-PKG signaling pathway**		
**Environmental Information Processing**	**Signal Transduction**	**Sphingolipid signaling pathway**		
**Environmental Information Processing**	**Signal Transduction**	**MAPK signaling pathway**		
**Environmental Information Processing**	**Signal Transduction**	**mTOR signaling pathway**		
**Environmental Information Processing**	**Signal Transduction**	**Wnt signaling pathway**		
Environmental Information Processing	Signal Transduction	Ras signaling pathway		
Environmental Information Processing	Signal Transduction	TGF-beta signaling pathway		
Environmental Information Processing	Signal Transduction	NF-kappa B signaling pathway		
Environmental Information Processing	Signal Transduction	AMPK signaling pathway		
Environmental Information Processing	Signal Transduction	Phosphatidylinositol signaling system		
Environmental Information Processing	Signal Transduction	Calcium signaling pathway		
**Environmental Information Processing**	**Signaling Molecules and Interaction**	**ECM-receptor interaction**		
Environmental Information Processing	Signaling Molecules and Interaction	Cytokine-cytokine receptor interaction		
Environmental Information Processing	Signaling Molecules and Interaction	Neuroactive ligand-receptor interaction		

‘Impact’ represents the change in the expression of the genes belonging to a specific pathway due to the supplementation of RPN; and ‘flux’ as the report of the average direction in the expression as downregulation, upregulation, or neutral or no change. Flux represents the direction of each subcategory belonging to ‘Environmental Information Processing’ KEGG category: green color represents inhibition, yellow neutrality, whereas red color shows activation. Color intensity depicts flux level. Blue lines show the impact of each category and the corresponding subcategory (*P* value < 0.05; FDR < 0.05). Subcategories and pathways highlighted in bold met the defined cutoff criteria.

The significant “Cellular processes” KEGG categories were ‘Cellular community–eukaryotes’, ‘Cell Growth and Death’, ‘Cell motility’, and ‘Transport and catabolism’. The “Cellular community–eukaryotes” KEGG subcategory showed two downregulated KEGG pathways: ‘Signaling pathways regulating pluripotency of stem cells’ and ‘Focal adhesion’. The “Cell Growth and Death” KEGG subcategory presented the downregulation of the ‘Apoptosis’ and ‘Cellular senescence’ KEGG pathways. The KEGG subcategory ‘Transport and catabolism’ had an inhibition of ‘Lysosome’ KEGG pathway (Table 4).

**Table 4:** Results of flux and impact uncovered by the Dynamic Impact Approach (DIA) based on Kyoto Encyclopedia of Genes and Genomes (KEGG) ‘Cellular Processes’ pathway database analysis of the bovine liver transcriptome of growing beef cattle supplemented with RPN.

KEGG category	KEGG subcategory	KEGG pathway	Impact	Flux
**Cellular Processes**	**Transport and Catabolism**	**Lysosome**		
Cellular Processes	Transport and Catabolism	Autophagy—animal		
Cellular Processes	Transport and Catabolism	Endocytosis		
Cellular Processes	Transport and Catabolism	Phagosome		
Cellular Processes	Transport and Catabolism	Peroxisome		
Cellular Processes	Transport and Catabolism	Mitophagy—animal		
**Cellular Processes**	**Cell Growth and Death**	**Apoptosis**		
**Cellular Processes**	**Cell Growth and Death**	**Cellular senescence**		
Cellular Processes	Cell Growth and Death	Necroptosis		
Cellular Processes	Cell Growth and Death	p53 signaling pathway		
Cellular Processes	Cell Growth and Death	Cell cycle		
Cellular Processes	Cell Growth and Death	Oocyte meiosis		
Cellular Processes	Cell Growth and Death	Ferroptosis		
**Cellular Processes**	**Cellular community—eukaryotes**	**Signaling pathways regulating pluripotency of stem cells**		
**Cellular Processes**	**Cellular community—eukaryotes**	**Focal adhesion**		
Cellular Processes	Cellular community—eukaryotes	Tight junction		
Cellular Processes	Cellular community—eukaryotes	Gap junction		
**Cellular Processes**	**Cell Motility**	**Regulation of actin cytoskeleton**		

‘Impact’ represents the change in the expression of the genes belonging to a specific pathway due to the supplementation of RPN; and ‘flux’ as the report of the average direction in the expression as downregulation, upregulation, or neutral or no change. Flux represents the direction of each subcategory belonging to ‘Cellular Processes’ KEGG category: green color represents inhibition, yellow neutrality, whereas red color shows activation. Color intensity depicts flux level. Blue lines show the impact of each category and the corresponding subcategory (*P* value < 0.05; FDR < 0.05). Subcategories and pathways highlighted in bold met the defined cutoff criteria.

The KEGG subcategories affected by RPN supplementation belonging to “Organismal System” KEGG category were ‘Immune system’, ‘Endocrine system’, ‘Digestive system’, ‘Development’ and ‘Aging’. The ‘Immune system’ KEGG subcategory showed the downregulation of the following KEGG pathways: ‘Fc epsilon RI signaling pathway’, ‘B cell receptor signaling pathway’, ‘Fc gamma R-mediated phagocytosis’, ‘T cell receptor signaling pathway’, ‘Toll-like receptor signaling pathway’, ‘Chemokine signaling pathway’, ‘IL-17 signaling pathway’, ‘RIG-I-like receptor signaling pathway’, ‘Leukocyte transendothelial migration’, ‘Neutrophil extracellular trap formation’, ‘Th17 cell differentiation’ and, ‘Platelet activation’. The “Digestive system” KEGG subcategory presented the downregulation of ‘Carbohydrate digestion and absorption’, ‘Protein digestion and absorption’ and, ‘Cholesterol metabolism’ KEGG pathways. Furthermore, ‘Prolactin signaling pathway’, ‘GnRH secretion’, ‘Thyroid hormone signaling pathway’, ‘Regulation of lipolysis in adipocytes’, ‘Relaxin signaling pathway’, ‘Growth hormone synthesis, secretion and action’, ‘Adipocytokine signaling pathway’, ‘Progesterone-mediated oocyte maturation’, ‘Parathyroid hormone synthesis, secretion and action’, ‘PPAR signaling pathway’, ‘Estrogen signaling pathway’ and, ‘Insulin signaling pathway’ were the downregulated KEGG pathways that belong to “Endocrine system” KEGG subcategory. The KEGG pathways downregulated in “Aging” KEGG subcategory were ‘Longevity regulating pathway—multiple species’ and ‘Longevity regulating pathway’. Finally, the ‘Osteoclast differentiation’ KEGG pathway that belongs to the “Development” KEGG subcategory was inhibited (Table 5).

**Table 5:** Results of flux and impact uncovered by the Dynamic Impact Approach (DIA) based on Kyoto Encyclopedia of Genes and Genomes (KEGG) ‘Organismal Systems’ pathway database analysis of the bovine liver transcriptome of growing beef cattle supplemented with RPN.

KEGG category	KEGG subcategory	KEGG pathway	Impact	Flux
**Organismal Systems**	**Immune System**	**Fc epsilon RI signaling pathway**		
**Organismal Systems**	**Immune System**	**B cell receptor signaling pathway**		
**Organismal Systems**	**Immune System**	**Fc gamma R-mediated phagocytosis**		
**Organismal Systems**	**Immune System**	**T cell receptor signaling pathway**		
**Organismal Systems**	**Immune System**	**Toll-like receptor signaling pathway**		
**Organismal Systems**	**Immune System**	**Chemokine signaling pathway**		
**Organismal Systems**	**Immune System**	**IL-17 signaling pathway**		
**Organismal Systems**	**Immune System**	**RIG-I-like receptor signaling pathway**		
**Organismal Systems**	**Immune System**	**Leukocyte transendothelial migration**		
**Organismal Systems**	**Immune System**	**Neutrophil extracellular trap formation**		
**Organismal Systems**	**Immune System**	**Th17 cell differentiation**		
**Organismal Systems**	**Immune System**	**Platelet activation**		
Organismal Systems	Immune System	NOD-like receptor signaling pathway		
Organismal Systems	Immune System	Th1 and Th2 cell differentiation		
Organismal Systems	Immune System	Complement and coagulation cascades		
Organismal Systems	Immune System	Antigen processing and presentation		
**Organismal Systems**	**Endocrine System**	**Prolactin signaling pathway**		
**Organismal Systems**	**Endocrine System**	**GnRH secretion**		
**Organismal Systems**	**Endocrine System**	**Thyroid hormone signaling pathway**		
**Organismal Systems**	**Endocrine System**	**Regulation of lipolysis in adipocytes**		
**Organismal Systems**	**Endocrine System**	**Relaxin signaling pathway**		
**Organismal Systems**	**Endocrine System**	**Growth hormone synthesis, secretion and action**		
**Organismal Systems**	**Endocrine System**	**Adipocytokine signaling pathway**		
**Organismal Systems**	**Endocrine System**	**Progesterone-mediated oocyte maturation**		
**Organismal Systems**	**Endocrine System**	**Parathyroid hormone synthesis, secretion and action**		
**Organismal Systems**	**Endocrine System**	**PPAR signaling pathway**		
**Organismal Systems**	**Endocrine System**	**Estrogen signaling pathway**		
**Organismal Systems**	**Endocrine System**	**Insulin signaling pathway**		
Organismal Systems	Endocrine System	Glucagon signaling pathway		
Organismal Systems	Endocrine System	Melanogenesis		
Organismal Systems	Endocrine System	GnRH signaling pathway		
Organismal Systems	Endocrine System	Oxytocin signaling pathway		
Organismal Systems	Endocrine System	Renin secretion		
Organismal Systems	Endocrine System	Thyroid hormone synthesis		
Organismal Systems	Endocrine System	Cortisol synthesis and secretion		
Organismal Systems	Endocrine System	Insulin secretion		
Organismal Systems	Endocrine System	Ovarian steroidogenesis		
Organismal Systems	Endocrine System	Aldosterone synthesis and secretion		
Organismal Systems	Circulatory System	Adrenergic signaling in cardiomyocytes		
Organismal Systems	Circulatory System	Vascular smooth muscle contraction		
**Organismal Systems**	**Digestive System**	**Carbohydrate digestion and absorption**		
**Organismal Systems**	**Digestive System**	**Protein digestion and absorption**		
**Organismal Systems**	**Digestive System**	**Cholesterol metabolism**		
Organismal Systems	Digestive System	Bile secretion		
Organismal Systems	Digestive System	Pancreatic secretion		
Organismal Systems	Digestive System	Salivary secretion		
Organismal Systems	Digestive System	Gastric acid secretion		
Organismal Systems	Excretory System	Vasopressin-regulated water reabsorption		
Organismal Systems	Excretory System	Endocrine and other factor-regulated calcium reabsorption		
Organismal Systems	Nervous System	Cholinergic synapse		
Organismal Systems	Nervous System	Neurotrophin signaling pathway		
Organismal Systems	Nervous System	Dopaminergic synapse		
Organismal Systems	Nervous System	Long-term depression		
Organismal Systems	Nervous System	Glutamatergic synapse		
Organismal Systems	Nervous System	Synaptic vesicle cycle		
Organismal Systems	Nervous System	Serotonergic synapse		
Organismal Systems	Nervous System	GABAergic synapse		
Organismal Systems	Nervous System	Long-term potentiation		
Organismal Systems	Nervous System	Retrograde endocannabinoid signaling		
Organismal Systems	Sensory System	Inflammatory mediator regulation of TRP channels		
Organismal Systems	Sensory System	Olfactory transduction		
**Organismal Systems**	**Development**	**Osteoclast differentiation**		
Organismal Systems	Development	Axon guidance		
**Organismal Systems**	**Aging**	**Longevity regulating pathway—multiple species**		
**Organismal Systems**	**Aging**	**Longevity regulating pathway**		
Organismal Systems	Environmental Adaptation	Circadian entrainment		
Organismal Systems	Environmental Adaptation	Thermogenesis		

‘Impact’ represents the change in the expression of the genes belonging to a specific pathway due to the supplementation of RPN; and ‘flux’ as the report of the average direction in the expression as downregulation, upregulation, or neutral or no change. Flux represents the direction of each subcategory belonging to ‘Organismal systems’ KEGG category: green color represents inhibition, yellow neutrality, whereas red color shows activation. Color intensity depicts flux level. Blue lines show the impact of each category and the corresponding subcategory (*P* value < 0.05; FDR < 0.05). Subcategories and pathways highlighted in bold met our cutoff criteria.

The “Biological Processes” GO terms affected by RPN supplementation were ‘GO:0006412~translation’ with 29 DEG (FDR = 0.01) and ‘GO:0055114~oxidation-reduction process’ with 41 DEG (FDR = 0.04) (S4 Table). The significant “Cellular Processes” GO terms were ‘GO:0070062~extracellular exosome’ with 188 DEG (FDR = 3.47 x 10^−4^), ‘GO:0005747~mitochondrial respiratory chain complex I’ with 14 DEG (FDR = 9.45 x 10^−4^) and ‘GO:0016020~membrane’ with 90 DEG (FDR = 0.001) (S5 Table). Finally, the significant “Molecular Function” GO terms were ‘GO:0003735~structural constituent of ribosome’ with 34 DEG (FDR = 0.001) and ‘GO:0044822~poly(A) RNA binding’ with 85 DEG (FDR = 0.008) (S6 Table).

## Discussion

### KEGG pathways

#### Metabolism

Arachidonic acid, a ω-6 polyunsaturated fatty acid, is present in the cytosol of the cells in a close spatial relationship with the endoplasmic reticulum membrane. This location allows arachidonic acid to interact with proteins involved in phospholipid synthesis [28]. The downregulation of “arachidonic acid metabolism” pathway could be in accordance with previous evidence showing the inhibitory effect of niacin on lipolysis, specifically of reduced secretion of VLDL molecules in humans [29]. One example of lipolysis disturbance could be explained the downregulation of 85 kDa calcium-independent phospholipase A2 gene (*PLA2G6*, logFC = -3.29; FDR = 0.04) in our study. The *PLA2G6* gene plays a significant role in phospholipid remodeling through catalysis of glycerophospholipid into arachidonic acid and a 2-lysophospholipid [30]. Since lipid accumulation in liver is detrimental, niacin supplementation has been utilized in humans as a pharmacological tool to decrease the flux of lipids from adipose tissue in order to reduce fatty liver conditions [31]; nevertheless, we did not measure the concentration of lipids in hepatic cells. Even though hepatic lipid accumulation does not represent a problem in growing beef cattle, RPN supplementation may improve liver health status by preventing hepatic lipid accumulation [32].

Furthermore, RPN supplementation led to a downregulation in “Other types of O-glycan biosynthesis” pathway. Glycans are defined as carbohydrates linked to proteins, forming glycoproteins; or in lipids, composing glycolipids. Glycans are involved in numerous cellular functions, such as protein folding or signaling, and glycosylation, which occurs as a posttranslational modification of proteins [33]. The glycosylation process starts by the addition of O-linked monosaccharide β-N-acetylglucosamine (GlcNAc) onto serine or threonine hydroxyl groups, which are included in the KEGG pathway “Other types of O-glycan biosynthesis” [34]. In our study, there was a downregulation of polypeptide N-acetylgalactosaminyltransferase 16 (*GALNT16*, logFC = -2.24, FDR = 0.04) and protein O-fucosyltransferase 2 (*POFUT2*, logFC = -2.26, FDR = 0.03), which are responsible for transferring N-acetyl-D-galactosamine and fucose to a serine or threonine residue, respectively [35, 36]. A possible explanation for the inhibition of these genes could be associated with the effect of rumen-protected niacin on hepatic apolipoproteins (apo) B belonging to VLDL and LDL, however, our study did not find significant changes in ApoB expression. It has been previously shown that glycation of human apo-Bs leads to lipidemia [37]. Results from our study demonstrate that RPN supplementation downregulates genes that encode for proteins of glycan biosynthesis, which might suggest that glycation could potentially be inhibited, leading to decreased VLDL and LDL synthesis [38]. Further research may elucidate changes in animal performance and carcass quality due to reduced circulating VLDL and LDL.

#### Cellular processes

The functions of niacin on protection from DNA damage and maintenance of genomic stability have been well investigated in humans and mice [39–41]. A lack of niacin in the human body can delay excision repair, and impair cell cycle arrest and apoptosis as a response to DNA damage [39]. Thus, the alteration of cellular senescence and apoptosis pathways in our results is not surprising. For example, studies have shown that niacin is associated with the alteration of apoptosis-inducing factor (AIF) translocation and apoptosis process in a caspase activation independent way, due to changes in poly (ADP-ribose) polymerase-1 (*PARP-1*) activity [41]. Another study in diabetic mice found that niacin can decrease the high glucose-induced reactive oxidative stress, cell apoptosis and senescence of endothelial progenitor cell in mice [41]. The downregulation in ‘Cell growth and death’ KEGG category occurred from the downregulation of cellular senescence and apoptosis pathways in our results and supports the previous findings of niacin functions.

As for the other significant pathways involved in cellular processes, it is worth mentioning the downregulation of cell motility and cell community KEGG categories, which mainly rely on the downregulation of the “regulation of actin cytoskeleton” pathway and “focal adhesion” pathway. A recent article investigating niacin deficiency and genetic instability also reported alterations of the focal adhesion signaling pathway in niacin-deficient cells in humans [42]. Focal adhesions are involved in various cellular processes, including migration, proliferation, and differentiation [43]. The physical linkage between the extracellular substrate and the actin cytoskeleton at focal adhesions is necessary for cell migration [44]. Our findings suggesting that RPN treatment leads to the downregulation of genes associated with focal adhesion [i.e., actinin (*ACTN4*, logFC = -2.54, FDR = 0.03), filamin (*FLNA*, logFC = -2.54, FDR = 0.03), talin (*TLN1*, logFC = -2.35, FDR = 0.03), and zyxin (*ZYX*, logFC = -2.23, FDR = 0.02)], actin cytoskeleton [i.e., myosin II (*MYH14*, logFC = -5.71, FDR = 0.01) and actin related protein 2/3 complex subunit 1B (*ARPC1B*, logFC = -5.71, FDR = 0.01)] can be explained in a way based on the lipid metabolism regulation function of niacin that we have discussed earlier. Niacin can prevent atherosclerotic cardiovascular disease, and oxidative modification of low-density lipoprotein (oxLDL), which is the major atherogenic modification of low-density lipoprotein [45]. A previous study found that oxLDL induced phosphorylation of focal adhesion kinase (p-*FAK*) and actin polymerization can be reduced by niacin in mice [46]. Furthermore, the enhancement of *FAK* phosphorylation and actin polymerization is associated with the inhibition of cell migration [47]. In addition, another study in vascular smooth muscle cells (VSMCs) revealed that niacin attenuated the oxLDL-induced apoptosis by inhibiting the *FAK* signaling pathway in mice [48]. Finally, the niacin receptor Hydroxycarboxylic acid receptor 2 (*HCA2*) is involved in cellular inflammatory responses. The chemokine-induced migration of macrophages can be inhibited by the niacin-activated *HCA2* [49], which also corresponds to our findings of the downregulation of the immune system.

#### Environmental information processing

Niacin, known to lower cytosolic NADH/NAD^+^ ratio, was shown to block the Janus kinase-signal transducers and activators of transcription (JAK-STAT) pathway which is activated via phosphorylation [50]. The inhibition of the JAK-STAT cascade, which is one of the major inflammatory pathways signaling downstream of cytokines, alters the recruitment of other molecules, or processes downstream signals via the Ras-Raf-MAP kinase and PI3 kinase pathways. In the liver, *JAK2* is activated by several cytokines and growth factors, including IFN-γ, IL-4, IL-6, IL-12, IL-13, growth hormone (GH), and leptin [51]. Signal transducer and transcription activators (STATs) mediate cellular responses to above mentioned chemical signals. Our results suggest that STATs did not experience phosphorylation by JAKs, because STATs dimerization was downregulated; therefore, STAT homodimer translocation to the nucleus could have not occur, inhibiting apoptosis and promoting liver regeneration [52].

Niacin mediates its anti-inflammatory effects via HCA2-dependent mechanisms in monocytes and macrophages [53, 54] by inhibiting their adhesion and accumulation in adipose tissue by oxLDL [55], and in vascular endothelium inhibiting angiotensin II-induced reactive oxygen species (ROS) production (Apelin Signaling Pathway).

Rumen-protected niacin increased the expression of tyrosine 3-monooxygenase/tryptophan 5-monooxygenase activation protein beta (*YWHAB*, logFC = 1.24, FC = 0.03) that belongs to the Hippo signaling pathway which is a critical regulator of liver size [56]. In a previous study, a peak of *YWHAB* expression plays critical roles in the termination of liver regeneration by inhibiting cellular proliferation in male rats [57], this is considered a suggested potential mechanism of *YWHAB* to control liver size. The decrease in organ size could be explained also by the lipolytic effect of niacin in liver adipose tissue content [31]. We were not able to measure liver size in our study. Also, in our study, axin-1 (*AXIN1*, logFC = - 2.19, FC = 0.03) and nucleoside diphosphate kinase 2 (*NKD2*, logFC = - 12.08, FC = 0.005) genes, which are known inhibitors of the β-catenin pathway, were inhibited. The antagonistic effect of *NKD2* on canonical Wnt signaling is achieved by inhibiting the translocation of β-catenin into the nucleus [58], which requires its interaction with *Axin2* [59], since both genes have antiapoptotic effects. The Hippo signaling pathway also presented signs of inhibition of macrophage polarization [60] in the liver through inhibition of *LLGL2* (logFC = -2.16, FDR = 0.01), and *SCRIB* (logFC = -3.08, FDR = 0.02) genes which is a typical reaction during pathological conditions.

The cAMP signaling pathway presented signs of nicotinic acid uptake and transport in hepatic tissue, which appears to be regulated by an intracellular Ca^2+^/calmodulin-mediated pathway in humans [61]. These signs were represented by the activation of ATPase plasma membrane Ca^2+^ transporting 1 (*ATP2B1*/*PMCA*, logFC = 1.71, FDR = 0.01) and calcium/calmodulin dependent protein kinase IV (*CAMK4*, logFC = 1.15, FDR = 0.04), in combination with inhibition of cAMP/PKA/CREB pathway, which is a major regulator of hepatic tissue proliferation and apoptosis [62]. Coincidentally with the WNT/Ca^2+^ pathway, the antilipolytic effect of nicotinic acid was evidenced by the Gi-mediated inhibition of adenylate cyclase 1, which inhibits cAMP production, through cholinergic receptor muscarinic 1 (CHRM1) activation [63].

The cGMP-PKG signaling pathway is mainly related to vascular contraction and relaxation. A vasodilator effect of RPN supplementation may have been observed during our study by the activation of natriuretic peptide receptor 2 (*NPR2*, logFC = 1.92, FDR = 0.02) and regulator of G protein signaling 2 (*RGS2*, logFC = 1.62, FDR = 0.01); both attenuate the increment of hepatic vascular resistance [64, 65]. Furthermore, myocyte enhancer factor 2 (*MEF2*, logFC = -2.75, FDR = 0.02) was inhibited by RPN supplementation. This gene has a role in the activation of hepatic stellate cells (HSCs), which represents a final common pathway of the hepatic response to liver injury [66]. Nevertheless, caution must been exercised when associating the effects of a physiologic phenomenon from a specific tissue to another, such as vasodilation in vascular tissue to hepatic tissue.

Our results suggested that RPN supplementation could potentially lead to hepatic glucose production *in vivo* through stimulation of a constitutively active Calcium/calmodulin-dependent protein kinase II (*CAMK2D*, logFC = 1.15, FDR = 0.04), which is activated in a calcium- and IP3R-dependent manner by cAMP and glucagon in primary hepatic tissue and by glucagon and fasting in vivo (WNT/Ca^2+^ pathway) [67]. Furthermore, niacin supplementation produces an inhibition of nuclear factor of activated T-cells, cytoplasmic 4 (*NFATC4*, logFC = -1.79, FDR = 0.02), which induces the expression of cytokine genes in T-cells, especially IL-2 or IL-4 [68].

Matrix metalloproteinase 14 (*MMP-14*, logFC = -3.51, FDR = 0.01) is associated with the degradation of several adhesion molecules, including fibronectin [69] which is the main component of the hepatic extracellular matrix. Particularly, *MMP14* participates in the remodeling of the extracellular matrix which could be harmful to the liver tissue by producing accumulation of extracellular matrix leading to scar tissue formation [69]. Genes that belong to the TNF signaling pathway suggested that RPN supplementation could potentially have a beneficial effect on this process because it reduces ECM deposition through inhibition of the fibrogenic cytokine transforming growth factor β 1 (*TGF-β1*, logFC = -5.32, FDR = 0.04) [70]. This statement can be supported by preliminary studies using pharmacologically relevant niacin concentrations to prevent stellate cell fibrosis (collagen type 1 inhibition) induced by *TGF-β1* or oxidative stress mediator hydrogen peroxide (H_2_O_2_), a major physiological stimulator of liver fibrosis [71].

Healthy adult hepatic tissue do not express Hedgehog ligands [72]. Hedgehog ligands undergo complicated posttranslational modifications that result in lipid attachment and multimerization within structure called exosomes in vertebrates [73]. Exosomes carry cargos, including lipid, RNA, and proteins, and play important roles in regulating various biological processes. The inhibition of genes that plays a role in the regulation of endosome-to-lysosome trafficking (i.e., *MEGF8*, logFC = -6.87, FDR = 0.01; *MGRN1*, logFC = -2.44, FDR = 0.01), which are connected to a key transmembrane protein involved in cell-cell communication system called smoothened (Smo). Smoothened is critical for embryonic development and adult tissue homeostasis in vertebrates [74].

Our results also suggest that RPN supplementation activates presenilin-2 (*PSEN2*, logFC = 1.51, FDR = 0.04), which is thought to regulate cell differentiation and survival [75], and inhibited *Deltex 2 (DTX2*, logFC = -1.89, FDR = 0.03), which is closely involved in cell growth, differentiation, apoptosis, as well as intracellular signal transduction by modulating the Notch signaling pathway [76].

Rumen-protected niacin supplementation produced an inhibition of the Phospholipase D (*PLD*) KEGG pathway. This was the clearest sign of the well-known vasodilator effect of niacin. Briefly, angiotensinogen (*AGT*, logFC = -1.94, FDR = 0.04), which causes vasoconstriction and regulates blood pressure was inhibited by RPN supplementation. Phospholipase C beta 2 (*PLCB2*, logFC = 1.44, FDR = 0.02) catalyzes the hydrolysis of 1-phosphatidylinositol 4,5-bisphosphate into diacylglycerol (DAG) and inositol 1,4,5-trisphosphate (IP3). This enzyme regulates the function of the endothelial barrier through intracellular signaling downstream of G protein-coupled receptors [77]. IP3 pathway stimulates sarcoplasmic reticulum release of calcium and activates protein kinase C (PK-C) via the formation of diacylglycerol (DAG), which stimulates contraction [78]. Therefore, upon niacin activation, *GPR109A* (also known as *HCAR2*) couples to a G(i) protein and inhibits adenylate cyclase activity (*ADCY5*, logFC = -3.83, FDR = 0.02) [79], leading to inhibition of liberation of free fatty acid and stimulating vasodilation by inhibiting *AGT*.

Niacin influenced VEGF signaling pathway by causing an inhibition on cell migration through actin reorganization and cell proliferation through the MAPK signaling pathway. A previous study showed that niacin interferes with the signaling cascade of chemoattractants in macrophages. More specifically, niacin might be inhibiting chemoattractant receptor activation that triggers actin cytoskeleton reorganization to form lamellipodia at the leading edge of macrophages. These are clear signs of the inhibitory effect of niacin on chemoattractant-induced cell migration [49] that leads to the macrophage proinflammatory responses of niacin that may contribute as a valuable therapeutic target.

Oxygen-sensing pathways, including the NOTCH-[80], PI3K-AKT-mTOR-[81], MAPK14-[81] and HIF1α-dependent pathways [82] have hypoxia-induced responses. Amongst these pathways, the best investigated oxygen responsive factor is the transcription factor hypoxia-inducible factor 1α (*HIF1A*). In our study, RPN supplementation caused an inhibition of inflammation (*ITGB4*, logFC = -5.72, FDR = 0.01) and increased activation of angiogenesis (*TEK*, logFC = 1.29, FDR = 0.04) observed in the HIF1 pathway. TEK receptor tyrosine kinase (*TEK*) is an inducible early response gene involved in hepatic tissue proliferation and liver regeneration [83].

Furthermore, the FOXO signaling pathway was downregulated, potentially inhibiting oxidative stress resistance and DNA repair (*GADD45*, logFC = -2.63, FDR = 0.02), glucose metabolism (*G6PD*, logFC = -5.36, FDR = 0.01) and immunoregulation (*KLF2*, logFC = -4.17, FDR = 0.03). In contrast, one of the autophagy-related genes was activated (*GABARAP*, logFC = 1.61, FDR = 0.005). Briefly, *GADD45* overexpression has been implicated in stress signaling in response to physiological or environmental stressors, which results in cell cycle arrest, DNA repair, cell survival and senescence, or apoptosis [84]. Glucose-6-phosphate dehydrogenase (*G6PD*), plays an important role in the production of NADPH and restoring the intracellular redox state in the setting of increased oxidant stress [85]. Although, the precise role of niacin on these processes needs to be elucidated. Furthermore, niacin-mediated inactivation of flow-induced transcription factor Krüppel-like factor 2 (*KLF2*) in endothelial cells results in reduced liver damage in mice [86]. Finally, autophagy is a lysosome-mediated catabolic process that targets cytosolic components to lysosomes to be degraded for the purposes of maintaining cellular homeostasis and supplying substrates for energy generation [87]. Dysregulation of autophagy is observed in animal models of diet-induced obesity, oxidative stress, and metabolic syndrome [88]. Our study showed potential signs of activation of this process due to the upregulation of *GABARAP*, which is responsible for the autophagy mechanism involving general membrane remodeling [89].

Our results indicate the downregulation of the ERBB signaling pathway (*PAK4*, logFC = -1.95, FDR = 0.03; *ELK1*, logFC = -1.74, FDR = 0.02), cell adhesion (*HRAS*, logFC = -3.66, FDR = 0.01; *ELK1*), protein synthesis (*AKT1/2*, logFC = -3.82, FDR = 0.03 and logFC = -9.36, FDR = 0.004, respectively; *ELK1*; *RPS6KB2*, logFC = -3.77, FDR = 0.008; *EIF4EBP1*, logFC = -2.75, FDR = 0.02), and cell survival (*AKT1/2; BAD*, logFC = -1.45, FDR = 0.04) in response to RPN supplementation. Briefly, p21-activated kinases (PAKs) mediate extracellular signals and regulate cell motility and morphology, cytoskeletal remodeling, cell proliferation, and apoptosis [90]. In terms of cell survival, *AKT* functions in an anti-apoptotic manner by directly phosphorylating the pro-apoptotic Bcl-2-associated death promoter (*BAD*) [91]. A prior study has shown that nicotinic acid infusion in rats results in dephosphorylation of *AKT* in insulin-sensitive tissues, such as liver. Furthermore, AKT/mTOR signaling pathway activation has a close relation with inflammation. Phosphorylation of *AKT* or *mTOR* can activate *NF-κB*, leading to its nuclear translocation, producing inflammatory cytokines, such as *INF-γ* and *TNF-α* [92]. This drives AKT/mTOR signaling to be a target of anti-inflammation. Thus, we hypothesized that the mechanism of anti-inflammatory effects of *HCAR2*, activated by RPN supplementation, took place through inhibiting AKT/mTOR signaling pathway in mice [93].

*RAP1* acts as a molecular switch that regulates the cell’s response (e. g., changes in orientation, cytoskeleton rearrangements) to external stimuli (i.e., mechanotransduction). Within the *RAP1* metabolic pathway, RPN inhibited the expression of guanine nucleotide exchange factor (*VAV2*, logFC = -4.45, FDR = 0.008), which plays an important role in angiogenesis. A previous study showed that niacin inhibits angiogenesis likely through cytoskeleton remodeling in humans [94]. In our study, FERM, ARH/RhoGEF and pleckstrin domain protein 2 (*FARP2*, logFC = -2.68, FDR = 0.02) has been inhibited by RPN. In addition, *FARP2* has a role in the actin cytoskeleton rearrangement of endothelial cells. Furthermore, the binding of integrins to their extracellular ligands is assisted by actin, producing an integrin–actin linkage mediated by talin 1 [95]. Talin 1 transitions integrins to an active state leading to cell adhesion, migration, or changes in polarity. Our results suggest that niacin also inhibited the conversion of mechanical forces into biochemical signals through this *RAP1* pathway, leading to attenuation of collagen accumulation exerting potential antifibrotic properties of niacin [70].

The sphingolipid signaling pathway shows a clear inhibition of apoptosis through the activation of phospholipase C beta 2 (*PLCB2*, logFC = 1.44, FDR = 0.02) and sphingosine-1-phosphate receptor 5 (*S1PR5*, logFC = 1.08, FDR = 0.04) and the inhibition of cathepsin D (*CTSD*, logFC = -2.47, FDR = 0.01), *AKT*, *MAPK11/15* (logFC = -2.13, FDR = 0.03 and logFC = -5.80, FDR = 0.005, respectively) and, BCL2 Associated X, Apoptosis Regulator (*BAX*, logFC = -3.26, FDR = 0.01). *PLCB2* regulates the function of the hepatic endothelial barrier and mediates intracellular signaling downstream of G protein-coupled receptors. In previous research, an increase in phospholipase C gamma 2 (*PLCγ2*) mRNA was detected in the late phase of rat liver regeneration [96]. Although, *TNF-α* alone cannot induce apoptosis in normal hepatic tissue, because TNF-α also activates antiapoptotic signal pathways. *TNF-α* induces *S1P* generation via sphingosine kinase 2 (*SPHK2*, logFC = -1.92, FDR = 0.03), which activates survival signals such as the PI3K/AKT pathway and protects human hepatic tissue from TNF-α-induced apoptosis [97]. Our results suggest that RPN supplementation could inhibit apoptosis in hepatic tissue through the activation of *S1PR5* and the inactivation of TNF receptor superfamily member 1A (*TNFRSF1A*, logFC = -2.23, FDR = 0.03), which provides instructions for making a tumor necrosis factor receptor 1 (*TNFR1*), and *SPHK2* that encodes one of two sphingosine kinase isozymes that catalyze the phosphorylation of sphingosine into sphingosine 1-phosphate (*S1P*). *S1P* mediates many cellular processes, including migration, proliferation, and apoptosis. Cell death mediated by *TNF-α* employs ceramide as an important second messenger. Ceramide is further hydrolyzed by ceramidase to sphingosine, which subsequently is converted to *S1P* by *SPHK2*. The balance between intracellular concentrations of ceramide and *S1P* may be a critical factor in the determination of cell fate, and our results suggest that RPN supplementation drives this reaction to greater *S1P* production leading to cell survival. Although, little is known about the signaling pathways regulated by ceramide in hepatic tissue [97].

### Organismal systems

Niacin is known to inhibit lipolysis [98], in particular a decreased secretion of VLDL particles is traditionally associated with niacin effect [38]. For this reason, it is important to highlight the downregulation of ‘Lipid metabolism’ in our experiment (S3 Fig), which was mainly relied on the downregulation of ‘Glycerolipid metabolism’ and ‘Glycerophospholipid metabolism’ pathways. Within these two pathways we notably detected the downregulation of Lipin 3 (*LPIN3*, logFC = -2.82, FDR = 0.02), diacylglycerol kinase theta (*DGKQ*, logFC = -3.04, FDR = 0.04), monoglyceride lipase (*MGLL*, logFC = -1.92, FDR = 0.02), and phosphatidylethanolamine-N-methyltransferase (*PEMT*, logFC = -4.08, FDR = 0.02) genes. In particular, the *PEMT* downregulation is remarkable, considering that PEMT pathway is known to play a role in lipid metabolism by regulating VLDL secretion in mice [99]. Furthermore, the downregulation of ‘Regulation of lipolysis in adipocytes’ pathway was compatible with the general scenario of lipolysis inhibition. Notably, within the ‘Regulation of lipolysis in adipocytes’ pathway, we detected the downregulation of protein kinase A, CAMP-activated non-catalytic subunit gamma 1 (*PRKAG1*, logFC = -1.43, FDR = 0.04), which appeared in line with the role of niacin in lipolysis inhibition via reduction of cAMP [98]. Mechanistically, a reduced protein kinase A (*PKA*) activation leads to a less phosphorylation and activation of the lipolytic enzymes [98]. In this regard, it is interesting to note that we detected the upregulation of protein phosphatase 2 regulatory subunit B’epsilon (*PPP2R5E*, logFC = 1.34, FDR = 0.04) which is known to inhibit AMP kinase [100]. Also, the downregulation of acetyl-CoA carboxylase alpha (*ACACA*, logFC = -2.42, FDR = 0.03) is in line within this context, considering its role in lipid metabolism [101]. Within ‘Lipid metabolism’ KEGG subcategory, we also detected the partial downregulation of ‘Steroid hormone biosynthesis’ pathway. This downregulation mainly relied on the inhibition of cytochrome P450, family 2, subfamily d, polypeptide 14 (*CYP2D14*, logFC = -2.42, FDR = 0.03), cytochrome P450 family 11 subfamily A member 1 (*CYP11A1*, logFC = -1.77, FDR = 0.04) and cytochrome P450 2D14-like (*MGC127055*, logFC = -2.55, FDR = 0.02). The inhibition of P450 enzymes by nicotinic acid has been long recognized [102]. Cytochromes P450 are a group of heme-thiolate monooxygenases found at highest concentrations in the liver, where are involved in an NADPH-dependent electron transport pathway [103], and oxidize a variety of structurally unrelated compounds, including steroids, fatty acids, and xenobiotics [104]. This was remarkable since niacin, collectively defined as nicotinamide and nicotinic acid [105], is converted to NAD and NADH, which serve not only as electron carriers in the well-known oxidative respiration [106] but are also important for nucleic acids, fatty acids, and cholesterol synthesis [107]. The marked upregulation of ‘Oxidative phosphorylation’ is consistent with the role of NAD and NADH as electron carriers. Its upregulation mainly relied on the upregulation of a cluster of NADH:ubiquinone oxidoreductase family genes (such as *NDUFA13*, logFC = 2.12, FDR = 0.01; *NDUFB1*, logFC = 1.86, FDR = 0.005, and *NDUFC1*, logFC = 2.23, FDR = 0.004) in our experiment and it is compatible with the niacin effect on boosting mitochondrial biogenesis and respiratory chain activity [108]. The fact that the ‘Mitochondrial respiratory chain complex I’ GO term resulted statistically significantly enriched in our experiment appeared in line with this (S6 Table).

Collectively, our results suggested from a transcriptomic point of view that niacin reduces hepatic triglyceride synthesis and increases hepatic lipid oxidation [31]. Furthermore, the downregulation of malonyl-CoA decarboxylase (*MLYCD*, logFC = -1.51, FDR = 0.04) and the upregulation of acyl-CoA synthetase l ong chain family member 4 (*ACSL4*, logFC = 1.52, FDR = 0.02), which are known to play an important role in the control of fatty acid oxidation [109, 110], were compatible with this depicted scenario.

It Is also noteworthy to highlight the downregulation of ‘Digestive System’ KEGG subcategory, mainly relying on the downregulation of ‘Cholesterol metabolism’, ‘Carbohydrate digestion and absorption’ and ‘Protein digestion and absorption’ pathways. In particular, the downregulation of ‘Cholesterol metabolism’ was compatible with known niacin effect on total cholesterol decreasing [111]. Within this pathway we notably detected the downregulation of Apolipoprotein E (*APOE*, logFC = - 4.93, FDR = 0.01), which is involved in many steps in lipid and lipoprotein homeostasis, for the triglyceride-rich lipoproteins and for HDL [112]. High expression levels of hepatic apoE are traditionally associated with an increase in VLDL triglyceride secretion [113]; thus, its downregulation appeared in line with the well documented effect of niacin in decreasing of hepatic synthesis of triglycerides and VLDL particles [114].

In this general context, the downregulation of ‘Bile secretion’ pathway seemed inconsistent with the increased biliary secretion traditionally associated with hypocholesterolaemic action of niacin [115]. However, within this pathway, we detected the upregulation of UDP glucuronosyltransferase family 1 member A6 (*UGT1A6*, logFC = 1.25, FDR = 0.03) and 3-hydroxy-3-methylglutaryl-CoA reductase (*HMGCR*, logFC = 1.57, FDR = 0.04) genes. UDP-glucuronosyltransferases (UGTs) are a superfamily of enzymes that generally fulfill detoxification roles, catalyzing the glucuronidation of various exogenous (i.e., environmental toxicants and dietary toxins) as well as endogenous compounds (i.e., bilirubin, steroid hormones, fat soluble vitamins) [116] to increase water-solubility and their elimination from the body in urine or bile [117]. Whereas the upregulation of *HMGCR* was particularly intriguing because of its role as rate-limiting enzyme for cholesterol synthesis [118]. Indeed, cell culture studies have shown that *AMPK* inactivates *HMGCR*, with a consequent inhibiting effect on cholesterol synthesis [119]. Thus, its upregulation is consistent with the downregulation of *PRKAG1* detected in our experiment. Nevertheless, the possible inhibition effect of nicotinic acid on *HMGCR* activity has been recently proposed [120], thus the role of niacin in regulating *HMGCR*, a key enzyme in cholesterol synthesis, would require further investigations in the light of evidence indicating its role as lipid-lowering molecule [38]. Although the ‘Cholesterol biosynthetic process’ was not enriched, within this BP GO term (S5 Table), along with *HMGCR*, we detected the downregulation of 3β-hydroxysteroid-Δ24 reductase (*DHCR24*, logFC = - 3.18, FDR = 0.03), the final enzyme of the cholesterol biosynthetic pathway [121] and *APOA4*, which is known to be involved in cholesterol transport [122]. At the same time, we detected the upregulation of lamin B receptor (*LBR*, logFC = 2.02, FDR = 0.004), whose protein exhibits sterol reductase activity essential for cholesterol biosynthesis [123].

The marked downregulation of ‘Protein digestion and absorption’ pathway was also intriguing. This mainly relied on the downregulation of several genes belonging to the collagen family group, such as *COL4A1* (logFC = - 7.7, FDR = 0.01), *COL6A1* (logFC = - 3.87, FDR = 0.02), *COL6A2* (logFC = - 3.78, FDR = 0.03), *COL18A1* (logFC = - 2.8, FDR = 0.02), which suggested and confirmed the role of niacin as anti-fibrotic agent, as previously shown in human liver [71].

The downregulation of the ‘Immune system’ category was also noteworthy. This result relied on marked downregulation of several pathways, such as ‘Platelet activation’, ‘Chemokine’, ‘Neutrophil extracellular trap formation’, ‘Th17 cell differentiation’ and ‘Fc gamma R-mediated phagocytosis’ pathways. Considering the effects of platelet activation on vasocontraction [124], our results were compatible with the well documented benefits exerted by niacin on vasodilation [125]. Within ‘Platelet activation’ pathway, we detected the downregulation of *AKT1*, Rho Guanine nucleotide-exchange factor (*ARHGEF1*, logFC = - 1.67, FDR = 0.04), *TLN1* and the simultaneous upregulation of Prostaglandin-Endoperoxide Synthase 1 (*PTGS1*, logFC = - 1.54, FDR = 0.01). Akt is a serine–threonine kinase that contributes to signaling and activation responses of platelets in mice [126]. Interestingly, *ARHGEF1* is known to be involved in platelet activity [127]. *TLN1* is required for platelet integrins activation [128]. Furthermore, the upregulation of *PTGS1* is noteworthy considering that prostaglandins induce vasodilation [129] and inhibit the aggregation of platelets [130]. The downregulation of ‘Chemokine signaling’ pathway was consistent with the nicotinic acid effect in the pro-atherogenic chemokines suppression [131, 132]. Furthermore, the downregulation of ‘Th17 cell differentiation’ pathway mainly due to *TGFB1* downregulation was in line with the documented inhibitory effect of niacin on *TGFB1* mRNA expression in hamster [133].

In this context, the downregulation of ‘Relaxin signaling’ pathway was interesting. Although originally known to be present in pregnant individuals, current research identified other biological functions of relaxin in both males and females with physiological roles in vasodilation [134], as shown in humans [135] and rats [136]. This result appeared in contradiction with the expected vasodilator effect of niacin. However, focusing on single DEGs involved in this pathway, we notably detected the downregulation of *TGFB1*. In this regard, the positive interaction of *TGFB1* with endothelin-1, a potent vasoconstrictor secreted by vascular endothelial cells, has been described [137]. Thus, we speculated about the possibility that the downregulation of *TGFB1* might indicate a vasodilation effect of niacin connected with the presumable decrease of endothelin levels. Also, the downregulation of ‘Vascular smooth muscle cells’ pathway was noteworthy, since this could be compatible with a vasodilation state. Within this pathway, we detected the downregulation of *AGT*, a precursor for angiotensin [138], which acts directly on vascular smooth muscle as a potent vasoconstrictor [139].

Overall, the downregulation of ‘Immune system’ KEGG category also supported the notion of a potential anti-inflammatory effect of niacin [140]. The downregulation of *TNFRSF1A*, which is known to be involved in ‘Adipocytokine signaling’ pathway, is in line with this idea. *TNFRSF1A* encodes for a protein called tumor necrosis factor receptor 1 (*TNFR1*) that, when attached to another protein called tumor necrosis factor (TNF), can trigger either inflammation or self-destruction of the cell (apoptosis).

Adipocytokines can influence many bioactivities: notably inflammatory processes, glucose and lipid metabolism [141]. Adiponectin, an important adipocytokine secreted by adipose tissue [142], deserves particular mention since niacin treatment is associated with an increase of adiponectin [143]. Adiponectin plays an important regulatory role in the energy metabolism of cell glucose, sugar, and fatty acids [144, 145], and participates in the regulation of cell proliferation [146], obesity [147], and immune function [148].

The downregulation of ‘Insulin signaling’ pathway was consistent with *AKT1* downregulation [149] and was intriguing. Indeed, the long-term effect of niacin treatment on the impairment of insulin sensitivity has been recently debated [98, 150]. In particular, the role of phosphodiesterase 3B on insulin resistance consequent to a long-term niacin treatment has been proposed [98]. Nevertheless, the downregulation of ‘Insulin signaling’ in our experiment could be compatible with an improved insulin sensitivity. Within ‘Insulin signaling’ pathway, we notably detected the downregulation of *G6PD*, which encodes for a key enzyme involved in the last step of gluconeogenic and glycogenolytic pathways, suggesting gluconeogenesis inhibition. It is well-known that insulin exerts control of gluconeogenesis by acting on the liver, but also by acting on other tissues [151]. For example, in pancreatic α cells, insulin inhibits the secretion of glucagon, which can indirectly lead to the suppression of hepatic glucose production by reducing hepatic glucagon signaling of mice [152]. This scenario was also supported in our experiment by the downregulation of ‘Glucagon signaling’ pathway, which suggested a normal glucose level and supports the hypothesis of an improved insulin sensitivity. Within this pathway the downregulation of CAMP responsive element binding protein 3 like 3 (*CREB3L3*, logFC = - 1.82, FDR = 0.02) is noteworthy, since CREB is known to be a key activator of the hepatic gluconeogenic gene regulation program [153]. Furthermore, considering that niacin treatment is associated with an increase of adiponectin, an important insulin-sensing hormone, we speculated about the possibility that the downregulation of ‘Insulin signaling’ detected in our experiment could be considered as a feedback effect of adiponectin increase, which was already suggested to play a role in improving insulin sensitivity [138, 154].

Lastly the downregulation of ‘Thyroid hormone’ pathway was compatible with the decrease of thyroid hormone levels associated with niacin supplementation [155]. In this regard, we notably detected the downregulation of thyroid hormone receptor alpha (*THRA*, logFC = - 2.46, FDR = 0.03). Furthermore, within this pathway we also detected the downregulation of Notch Receptor 1 (*NOTCH1*, logFC = - 4.51, FDR = 0.02). The lower expression of *NOTCH1* due to niacin treatment was already reported in an *in vitro* study on niacin effect on vascular inflammation inhibition in cattle [156].

## Conclusion

Rumen-protected niacin supplementation on growing steers and heifers for 30 days after weaning presented a significant list of potential benefits observed at the liver transcriptomics level. Several metabolic pathways revealed positive effects of administration of rumen-protected niacin on beef calves after weaning. The most impacted pathways showed that rumen-protected niacin had a down-regulatory effect on the expression of genes related to lipolysis, apoptosis, inflammatory responses, atherosclerosis, oxidative stress, and fibrosis, and enhancing vasodilation. Therefore, results from our study could potentially promote supplementation of rumen-protected niacin on beef cattle backgrounding operations or new arrivals to a feedlot, especially during the acclimation period when health status is often compromised. Although, a performance test with a greater number of animals should be conducted in order to confirm these results. Finally, it is important to remark that our study seeks to bring light on the specific role of niacin in growing beef cattle, and caution must be exercised when translating our findings to other species or cattle breeds (i.e., transition dairy cows).

## Supporting information

S1 Fig (TIF)

S2 Fig (TIF)

S3 Fig (TIF)

S4 Fig (TIF)

S5 Fig (TIF)

S1 Table Chemical composition of diet fed to growing beef cattle. (DOCX)

S2 Table Summary of RNA-seq yield, quality control, and alignment percentages. (DOCX)

S3 Table List of primers used for qRT-PCR validation assay. (DOCX)

S4 Table (XLSX)

S5 Table (XLSX)

S6 Table (XLSX)
