# Correction: Hepatic transcript profiling in beef cattle: Effects of rumen-protected niacin supplementation

**DOI:** 10.1371/journal.pone.0317922

**Published:** 2025-01-16

**Authors:** The PLOS ONE Staff

The Tables 1 to 5 are incorrectly formatted and should have been labelled as Figs 1 to 5. The authors have provided a corrected version of Figs 1 to 5 here.

**Fig 1:**
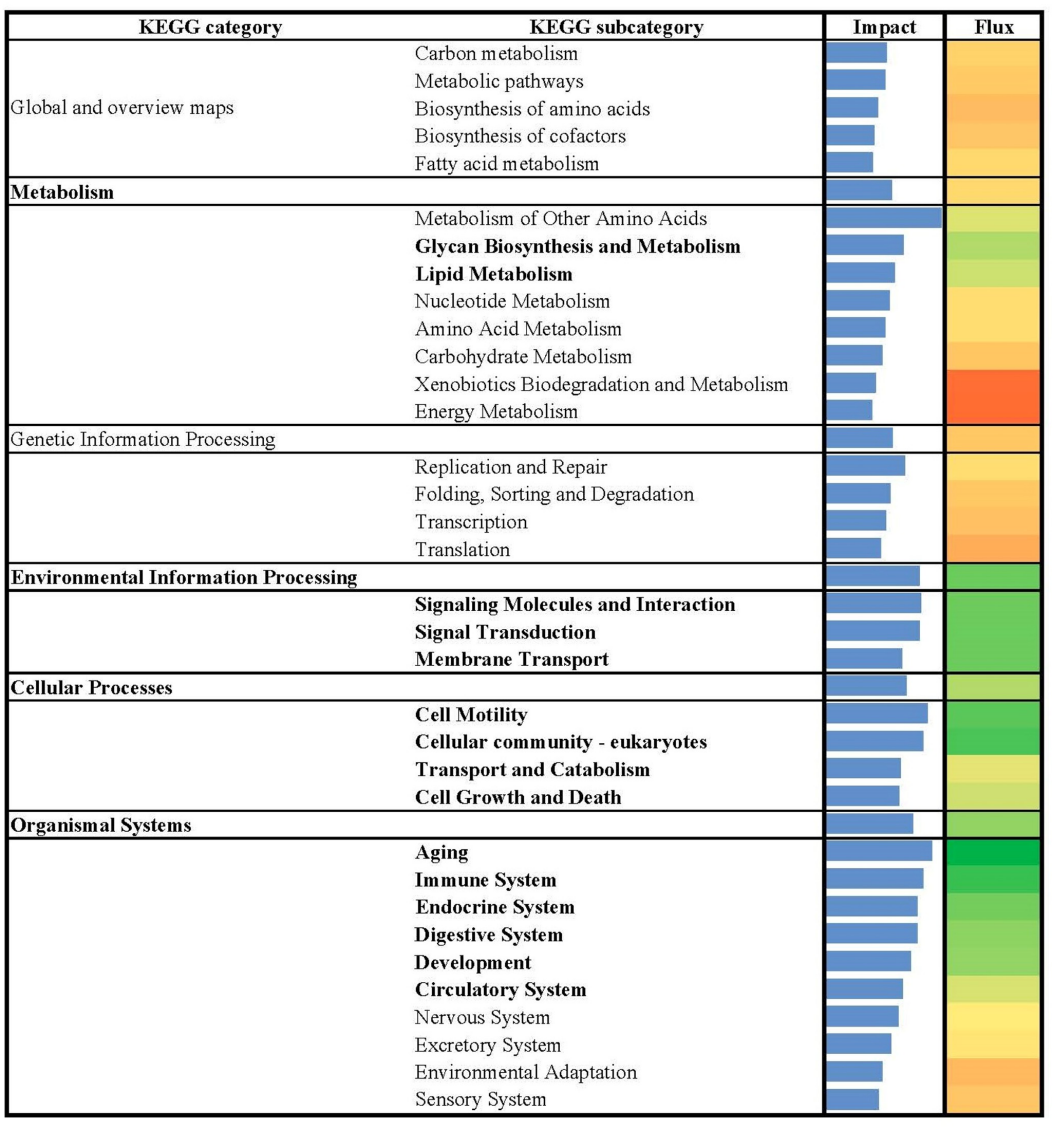
Summary of flux and impact results identified by the dynamic impact approach (DIA) based on Kyoto Encyclopedia of Genes and Genomes (KEGG) pathways databases analysis of the bovine liver transcriptome of growing beef cattle supplemented with or without RPN.

**Fig 2:**
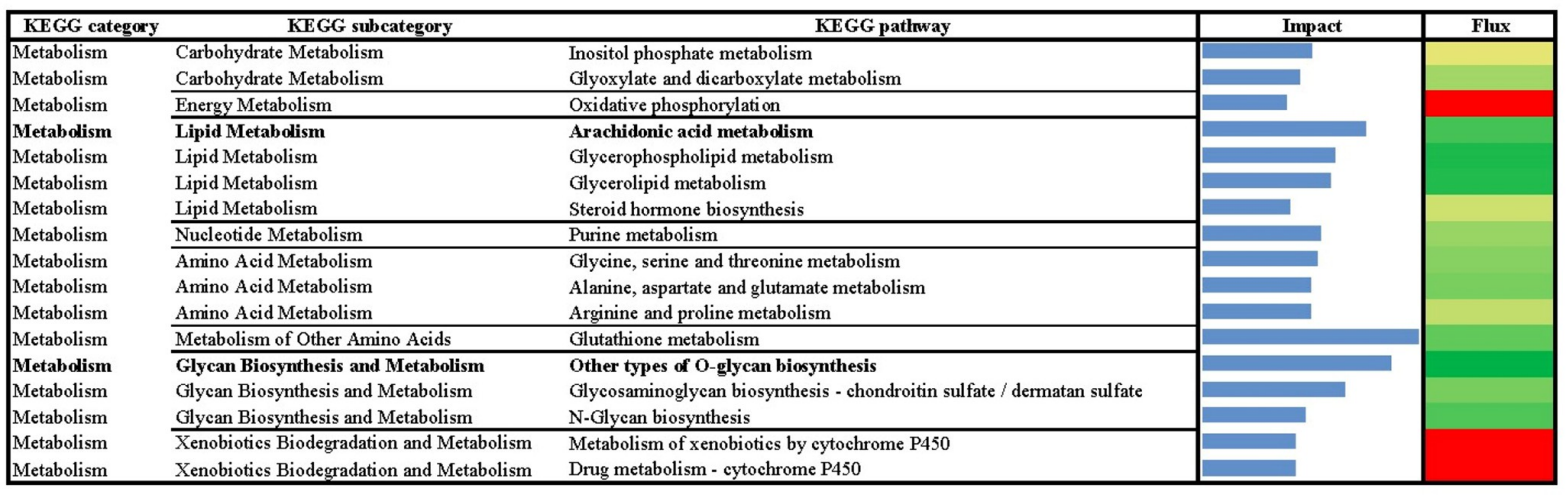
Results of flux and impact uncovered by the Dynamic Impact Approach (DIA) based on Kyoto Encyclopedia of Genes and Genomes (KEGG) ‘Metabolism’ category database analysis of the bovine liver transcriptome of growing beef cattle with or without RPN supplementation.

**Fig 3:**
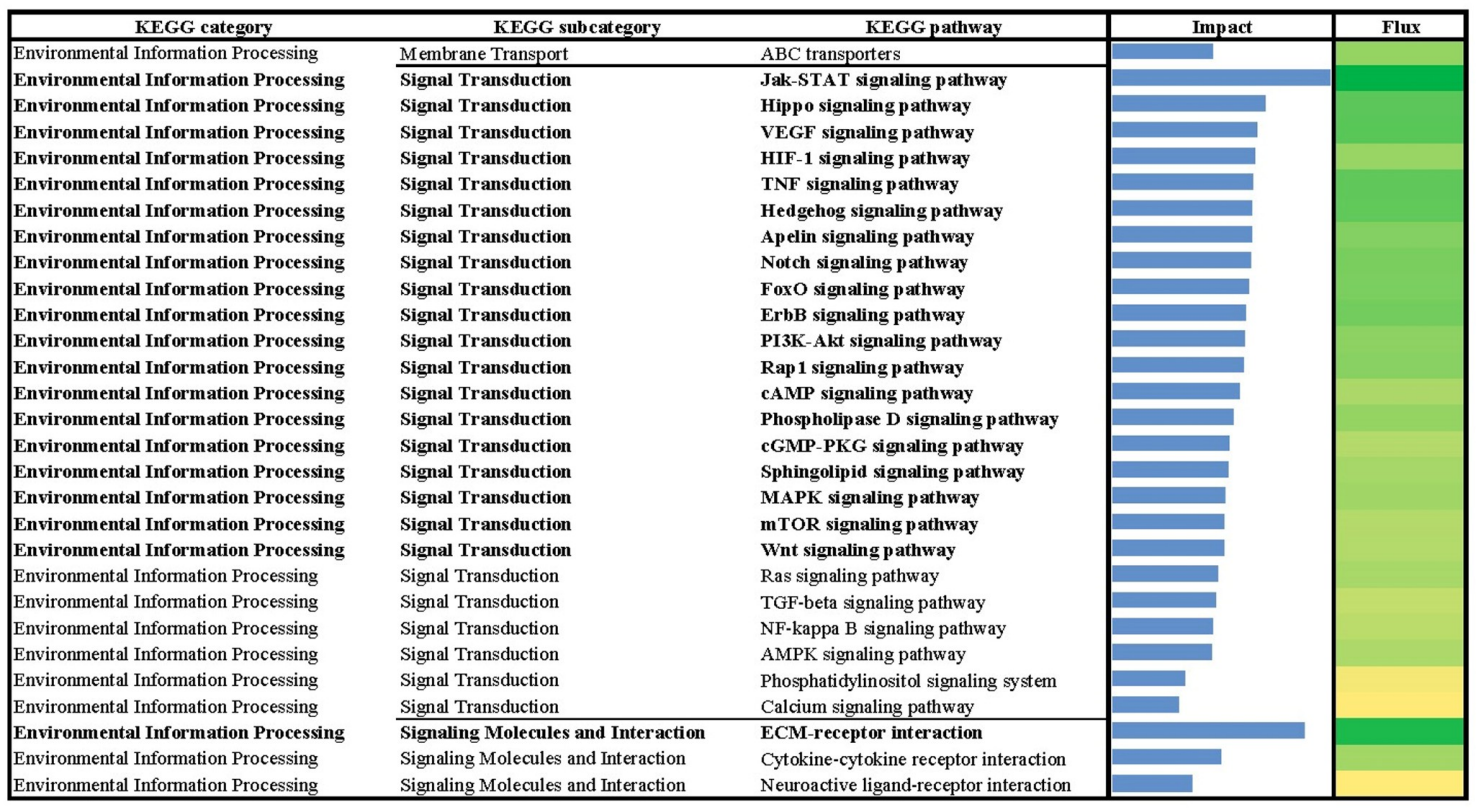
Results of flux and impact uncovered by the Dynamic Impact Approach (DIA) based on Kyoto Encyclopedia of Genes and Genomes (KEGG) ‘Environmental information processing’ pathway database analysis of the bovine liver transcriptome of growing beef cattle supplemented with RPN.

**Fig 4:**
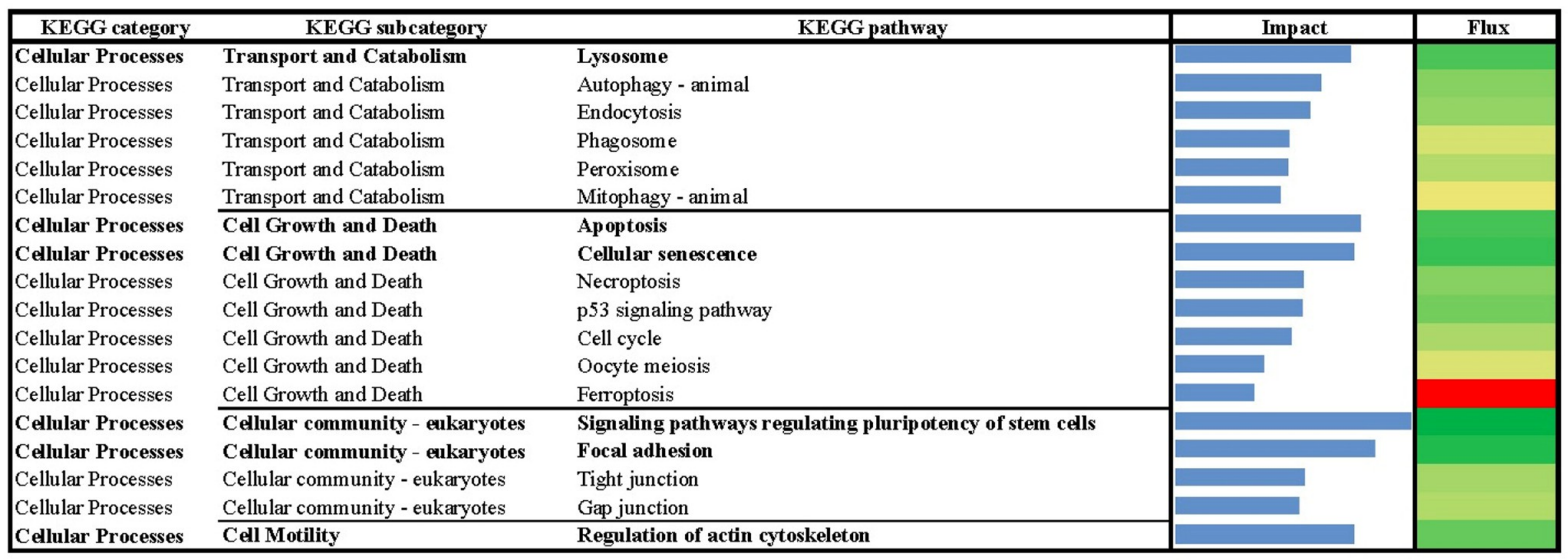
Results of flux and impact uncovered by the Dynamic Impact Approach (DIA) based on Kyoto Encyclopedia of Genes and Genomes (KEGG) ‘Cellular Processes’ pathway database analysis of the bovine liver transcriptome of growing beef cattle supplemented with RPN.

**Fig 5:**
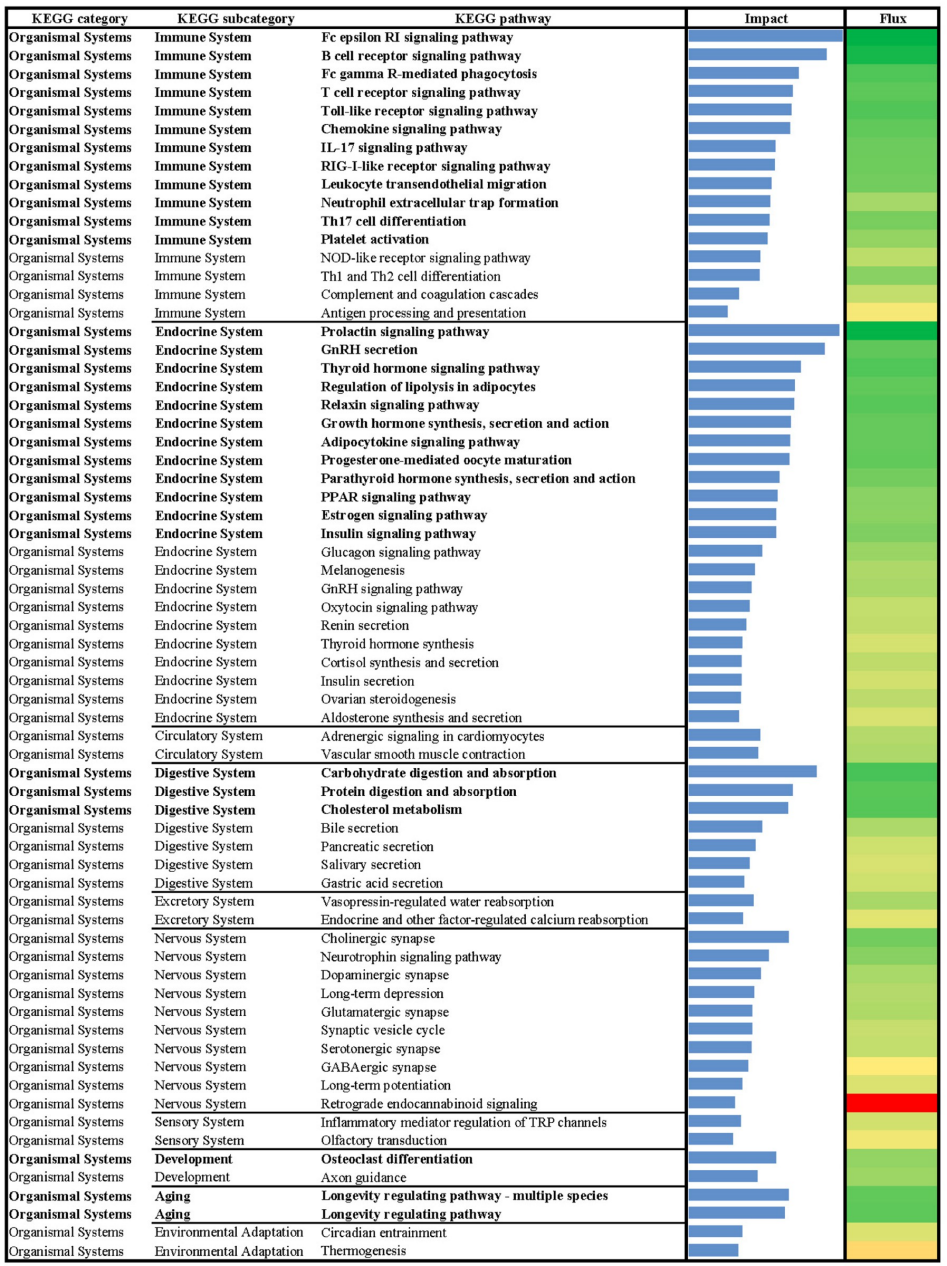
Results of flux and impact uncovered by the Dynamic Impact Approach (DIA) based on Kyoto Encyclopedia of Genes and Genomes (KEGG) ‘Organismal Systems’ pathway database analysis of the bovine liver transcriptome of growing beef cattle supplemented with RPN.

The publisher apologizes for the errors.
