# Supplementary figures and images for: Hepatic transcript profiling in beef cattle: Effects of rumen-protected niacin supplementation
Source: PLoS One. 2023 Aug 3;18(8):e0289409. doi: 10.1371/journal.pone.0289409 (PMC10399858; doi:10.1371/journal.pone.0289409)

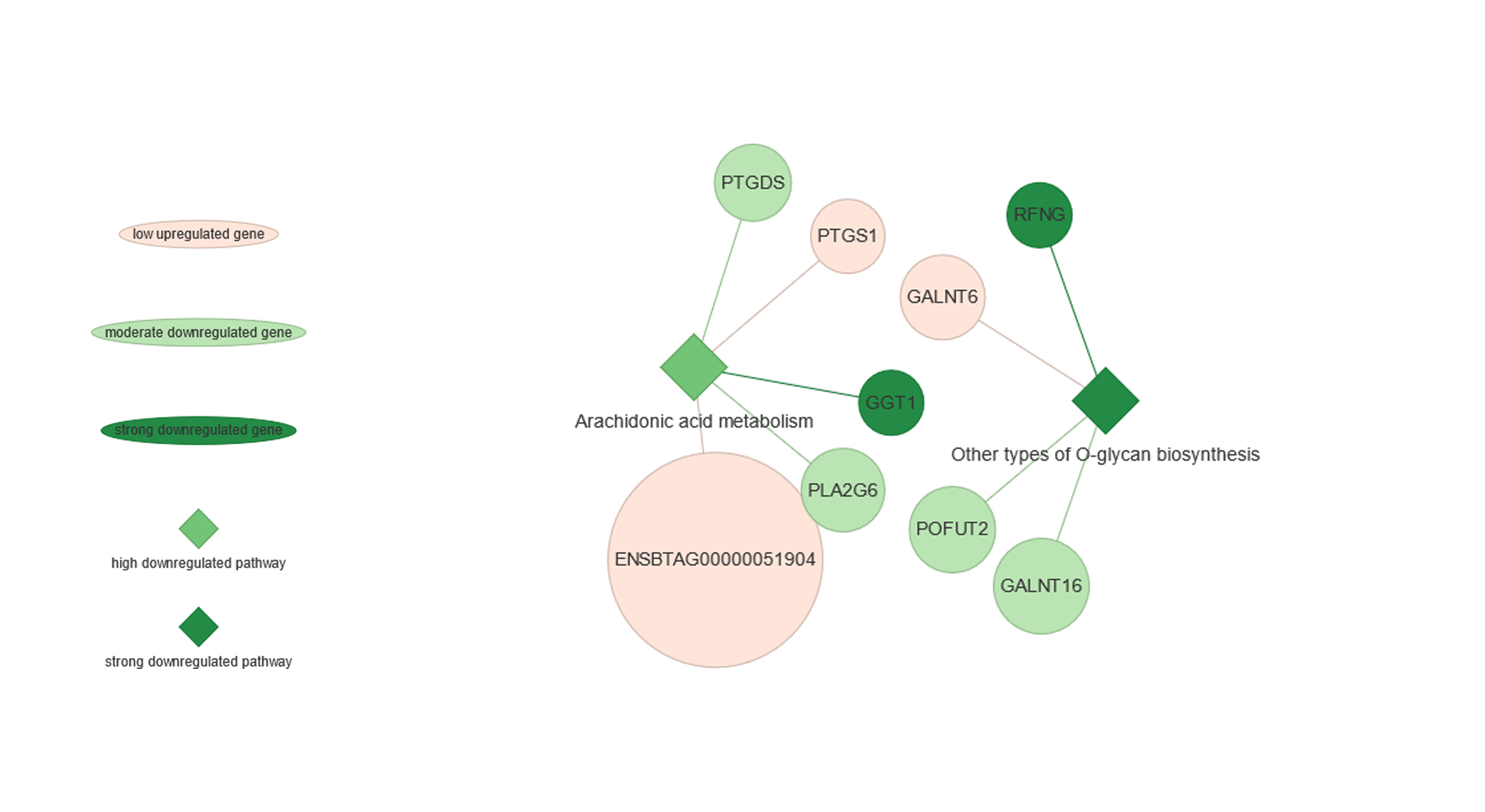

Supplement: S1 Fig — (TIF) [file pone.0289409.s001.tif]

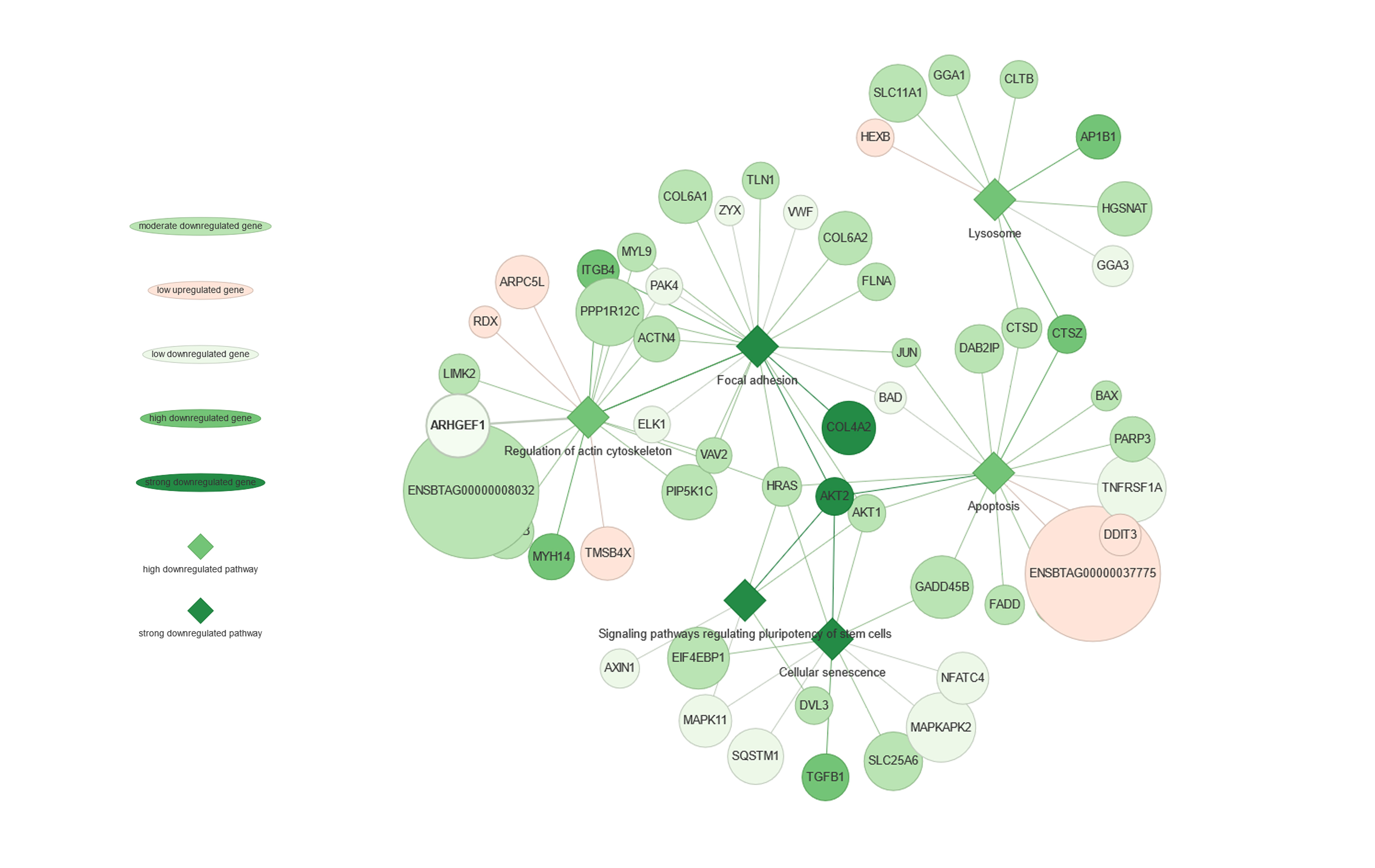

Supplement: S2 Fig — (TIF) [file pone.0289409.s002.tif]

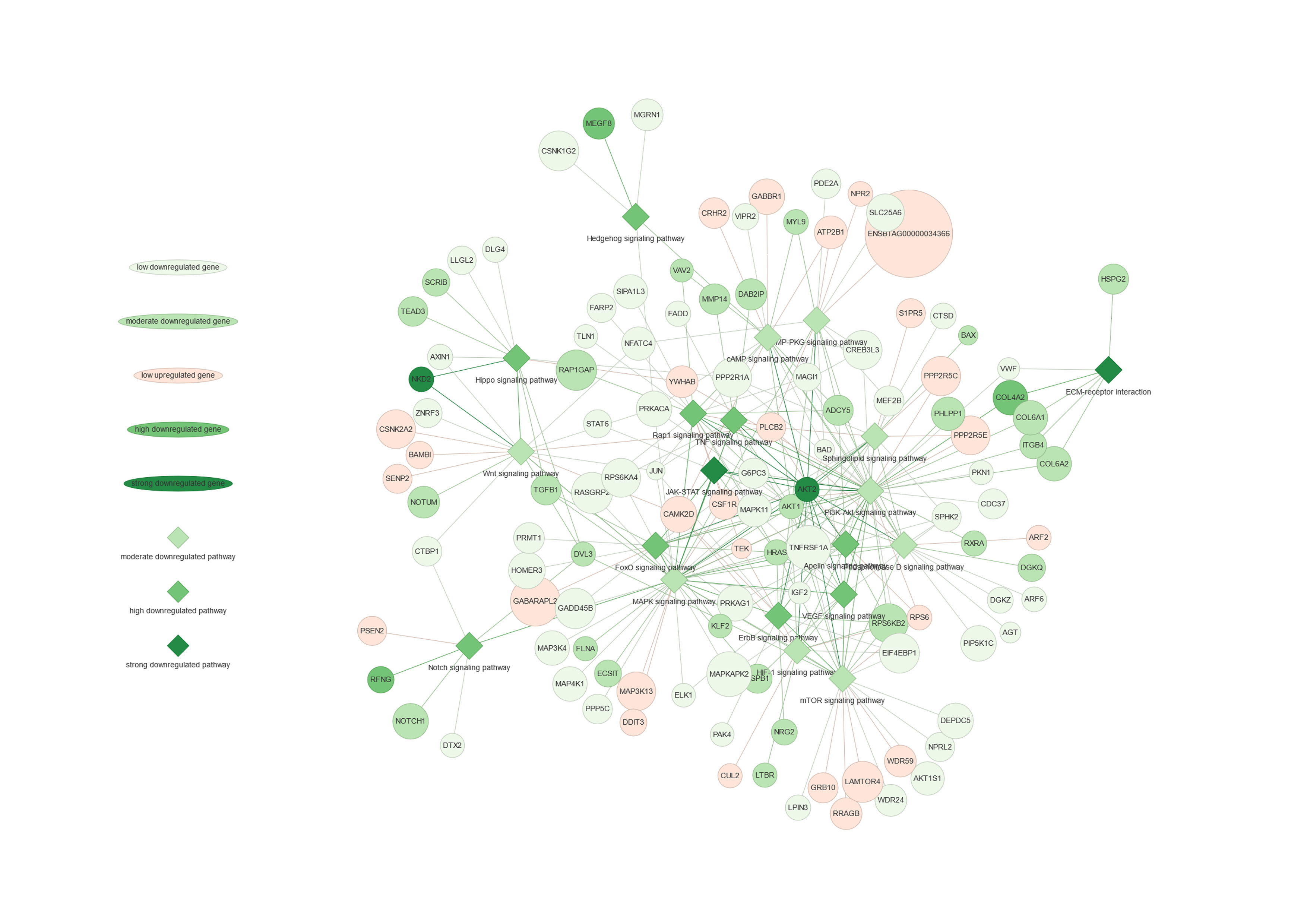

Supplement: S3 Fig — (TIF) [file pone.0289409.s003.tif]

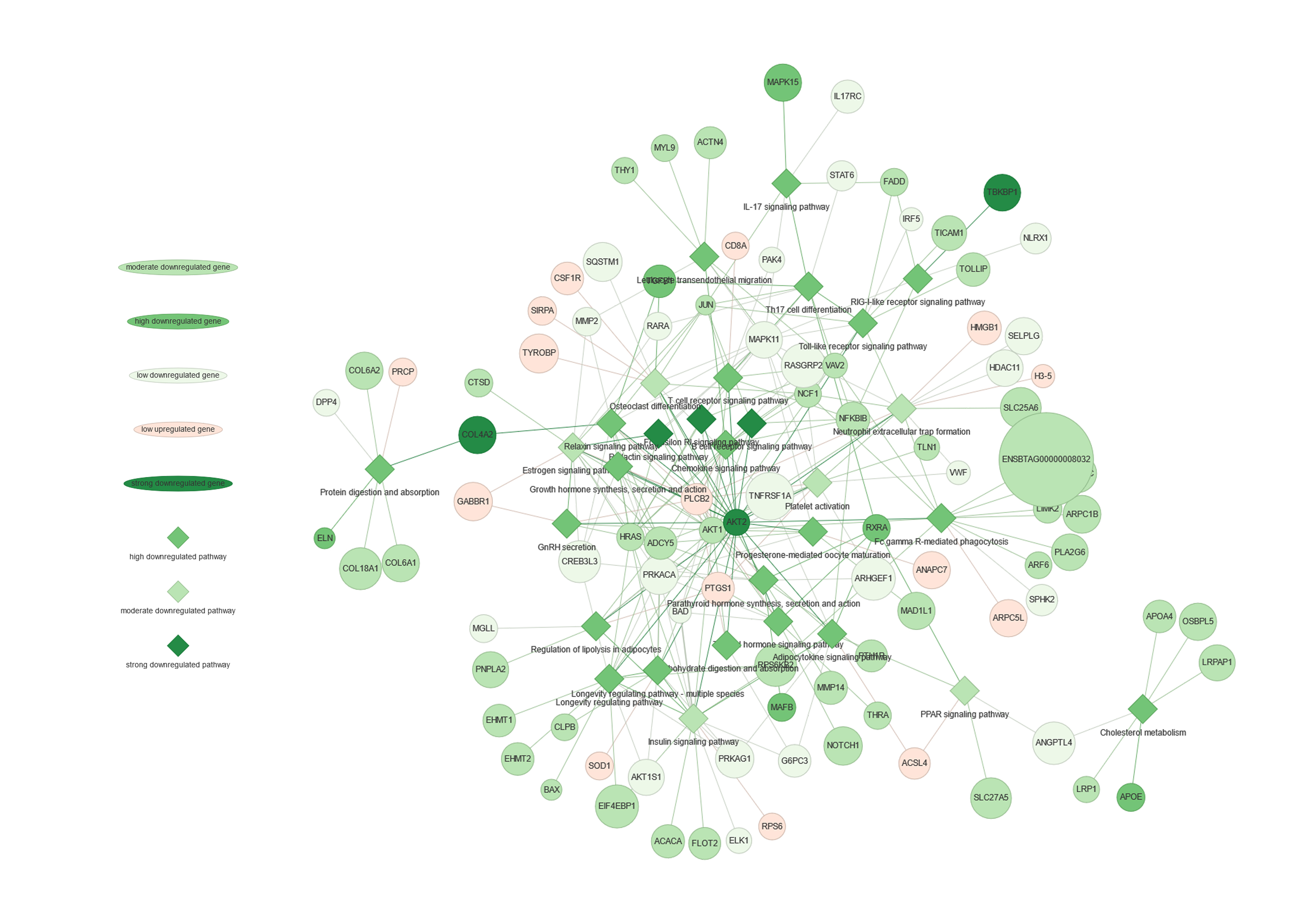

Supplement: S4 Fig — (TIF) [file pone.0289409.s004.tif]

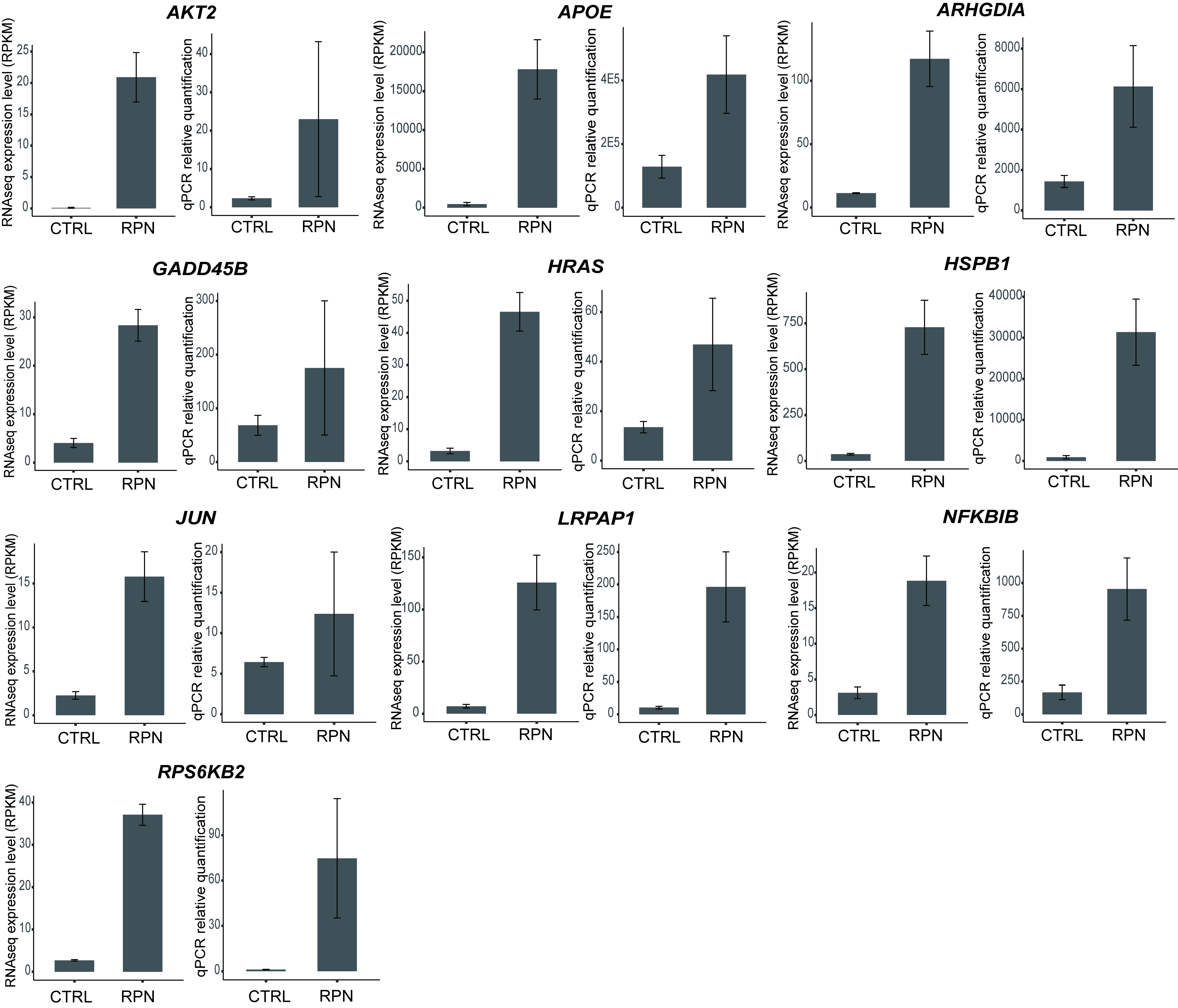

Supplement: S5 Fig — (TIF) [file pone.0289409.s005.tif]
