# Supplementary material for: Hepatic transcript profiling in beef cattle: Effects of rumen-protected niacin supplementation
Source: PLoS One. 2023 Aug 3;18(8):e0289409. doi: 10.1371/journal.pone.0289409 (PMC10399858; doi:10.1371/journal.pone.0289409)
Supplement: S1 Table — (DOCX) [file pone.0289409.s006.docx]

**Table S1.** Chemical composition of diet fed to growing beef cattle.

| **Ingredients** | **% DM** | **CP** | **NDF** | **ADF** | **TDN** | **Crude Fat** |
| --- | --- | --- | --- | --- | --- | --- |
| Fescue Seeds ^1^ | 90.55 | 16.12 | 48.49 | 16.03 | 64.11 | - |
| Pellets ^1,2^ | 90.59 | 29.63 | 12.29 | 4.30 | 74.45 | 1.84 |
| Molasses ^2^ | 84.00 | 5.80 | - | 0.40 | 72.00 | - |
| Bermudagrass hay | 84.90 | 14.36 | 31.65 | 63.87 | 64.55 | - |

^1^ Fescue seeds, pellets, and molasses were fed as supplement on a 48.5:48.5:3 ratio.

^2^ Pellets were composed by 46.5% ground corn, 46.5% soybean meal, 5% wheat middlings, and 2% soybean oil.

Bermudagrass hay was fed ad libitum, whereas 1.46 kg of fescue seeds, 1.46 kg of pellets. and 0.07 kg of molasses were offered individually in daily basis. All ingredients are expressed in a DM basis.
