# Supplementary material for: Hepatic transcript profiling in beef cattle: Effects of rumen-protected niacin supplementation
Source: PLoS One. 2023 Aug 3;18(8):e0289409. doi: 10.1371/journal.pone.0289409 (PMC10399858; doi:10.1371/journal.pone.0289409)
Supplement: S2 Table — (DOCX) [file pone.0289409.s007.docx]

**Table S2.** Summary of RNA-seq yield, quality control, and alignment percentages

| **Sample name** | **# of reads** | **# of reads after trimming** | **Trim surviving %** | **Mapped reads** | **Mapping %** |
| --- | --- | --- | --- | --- | --- |
| 9044G_NCNM_0716 | 143836264 | 141795772 | 98.58% | 128766947 | 90.80% |
| 9043G_NCNF_0716 | 151689854 | 150410714 | 99.16% | 140202620 | 93.20% |
| 9028G_NCNM_0716 | 80323338 | 79514228 | 98.99% | 72337769 | 91% |
| 9036G_TNCM_0716 | 82169668 | 80461072 | 97.92% | 72578322 | 90.20% |
| 9056G_TNCF_0716 | 89534924 | 86789500 | 96.93% | 66538273 | 76.70% |
| 9120G_TNCM_0716 | 80666926 | 78998438 | 97.93% | 68225659 | 86.40% |
